# Supplementary material for: Membrane-wide screening identifies potential tissue-specific determinants of SARS-CoV-2 tropism
Source: PLoS Pathog. 2025 Jul 17;21(7):e1013157. doi: 10.1371/journal.ppat.1013157 (PMC12286382; doi:10.1371/journal.ppat.1013157)
Supplement: S1 Table — (PDF) [file ppat.1013157.s001.pdf]

Supplementary Table 1.
Clinical cohort characteristics.

	**Number in cohort (N = 234,524)**
**COVID-19 hospitalized**	2,828 (1.21%)
**Age: mean (std)**	75.13 (9.35)
<= 20	0 (0.00%)
20–45	1,698 (0.72%)
45–65	19,853 (8.47%)
65–85	181,537 (77.40%)
> 85	31,436 (13.40%)
**Gender**	
Female	135,497 (57.78%)
Male	99,027 (42.22%)
**Race**	
White	154,704 (65.97%)
Black/African-American	35,096 (14.96%)
Asian	16,587 (7.07%)
Other	14,047 (5.99%)
Hispanic/Latinx	8,483 (3.62%)
Unknown	5,436 (2.32%)
Native American/American Indian/Alaska Native	171 (0.07%)
**Comorbidity Indices**	
Hypertension	170,502 (72.70%)
Diabetes without Chronic Complications	81,040 (34.56%)

Anemia Deficiency	48,625 (20.73%)
Diabetes with Chronic Complications	46,866 (19.98%)
Hypothyroidism	44,515 (18.98%)
Peripheral Vascular Disease	41,789 (17.82%)
Chronic Pulmonary Disease	39,795 (16.97%)
Obesity	30,911 (13.18%)
Cerebrovascular Disease	29,319 (12.50%)
Other Neurological Disorders	26,783 (11.42%)
Valvular Disease	26,660 (11.37%)
Solid Tumor without Metastasis	26,226 (11.18%)
Renal Failure	25,988 (11.08%)
Congestive Heart Failure	24,642 (10.51%)
Fluid and Electrolyte Disorders	19,665 (8.39%)
Depression	16,778 (7.15%)
Dementia	15,181 (6.47%)
Liver Disease	13,563 (5.78%)
Rheumatoid Arthritis	11,035 (4.71%)
Weight Loss	8,597 (3.67%)
Psychoses	6,789 (2.89%)
Myocardial Infarction	6,590 (2.81%)
Coagulopathy	5,797 (2.47%)
Paralysis	4,540 (1.94%)
Blood Loss Anemia	3,828 (1.63%)
Metastatic Cancer	3,079 (1.31%)
Pulmonary Circulation Disorder	2,748 (1.17%)

Peptic Ulcer Disease	2,625 (1.12%)
Drug Abuse	2,457 (1.05%)
Alcohol Abuse	2,450 (1.04%)
Lymphoma	2,202 (0.94%)
AIDS/HIV	973 (0.41%)
Moderate to Severe Liver Disease	701 (0.30%)
**Top 5 first 3-digits diagnosis codes**	
e78 (d/o lipoprotein metab lipidemias)	153,505 (65.45%)
m25 (other joint disorder nec)	54,267 (23.14%)
h25 (age-related cataract)	54,498 (23.24%)
m79 (other soft tissue disorders nec)	53,014 (22.60%)
m54 (dorsalgia)	48,682 (20.76%)
**Top 5 AHFS drug therapeutic classes**	
HMG-CoA reductase inhibitors	116,498 (49.67%)
Beta-adrenergic blocking agents	71,942 (30.68%)
Dihydropyridines	55,659 (23.73%)
Angiotensin II Receptor Antagonists*	53,098 (22.64%)
Angiotensin-Converting Enzyme Inhibitors*	47,879 (20.42%)
**Other features**	
Provider Visit in 2019	205,063 (87.44%)
Primary Care Provider Visit Count ≥5 in 2019	97,313 (41.49%)
Primary Care Provider Visit Count ≥11 in 2019	22,841 (9.74%)
3 Routine Lab Tests in 2019	136,964 (58.40%)
Inpatient Stay in 2019	27,653 (11.79%)
Flu Vaccination in 2019	140,760 (60.02%)

Count of Unique Drugs ≥6	132,974 (56.70%)
Count of Unique Drugs ≥10	58,215 (24.82%)
Count of Unique Drugs ≥14	21,962 (9.36%)
Special Needs Plan (SNP): Dual Plan	8,371 (3.57%)
SNP: Institutional	10,221 (4.36%)
SNP: Chronic	391 (0.17%)
