# Supplementary material for: Membrane-wide screening identifies potential tissue-specific determinants of SARS-CoV-2 tropism
Source: PLoS Pathog. 2025 Jul 17;21(7):e1013157. doi: 10.1371/journal.ppat.1013157 (PMC12286382; doi:10.1371/journal.ppat.1013157)
Supplement: S2 Table — (PDF) [file ppat.1013157.s002.pdf]

Supplementary Table 2.
Drugs most strongly associated with COVID-19 hospitalization in the database-wide screen

Generic Name	Drug User	Odds Ratio (95% CI)	Corrected p-value	AHFS Therapeutic Class
ipratropium/albuterol sulfate	3277	4.250 (3.944 - 4.581)	0	antimuscarinics /antispasmodics
divalproex sodium	1363	6.446 (5.783 - 7.185)	5.84E-246	anticonvulsants misc
mirtazapine	3183	3.708 (3.432 - 4.005)	2.32E-240	antidepressants misc
lactulose	1098	5.651 (5.006 - 6.379)	2.45E-170	ammonia detoxicants
trazodone hcl	4707	2.471 (2.304 - 2.650)	1.10E-139	serotonin modulators
oseltamivir phosphate	1369	4.126 (3.687 - 4.617)	1.99E-132	neuraminidase inhibitor antivirals
memantine hcl	2395	3.091 (2.824 - 3.384)	1.54E-129	central nervous system agents misc
levetiracetam	1370	4.054 (3.621 - 4.537)	1.26E-128	anticonvulsants misc
donepezil hcl	3695	2.542 (2.353 - 2.746)	1.69E-121	parasympathomimetic (cholinergic agents)
insulin lispro	3620	2.514 (2.325 - 2.719)	5.81E-116	rapid-acting insulins
nystatin	2969	2.697 (2.479 - 2.934)	1.47E-115	polyenes (skin and mucous membrane)
furosemide	13667	1.741 (1.657 - 1.829)	8.38E-105	loop diuretics
quetiapine fumarate	1928	3.001 (2.713 - 3.319)	3.48E-99	atypical antipsychotics
insulin glargine hum.rec.anlog	4811	2.074 (1.930 - 2.229)	1.17E-85	long-acting insulins
ondansetron hcl	2545	2.531 (2.309 - 2.774)	1.79E-85	5-ht3 receptor antagonists
gabapentin	10730	1.699 (1.610 - 1.794)	5.98E-80	anticonvulsants misc

famotidine	3594	2.145 (1.977 - 2.327)	2.19E-73	histamine h2-antagonists
sertraline hcl	4495	1.941 (1.800 - 2.093)	1.43E-64	selective-serotonin reuptake inhibitors
carbidopa/levodopa	1131	2.910 (2.553 - 3.316)	1.64E-55	dopamine precursors
lorazepam	2834	2.087 (1.905 - 2.287)	4.62E-54	benzodiazepines (anxiolytic sedativ/hyp)
tamsulosin hcl	7436	1.653 (1.552 - 1.761)	6.51E-53	selective alpha-1-adrenergic block.agent
escitalopram oxalate	3686	1.860 (1.711 - 2.021)	3.94E-46	selective-serotonin reuptake inhibitors
baclofen	1464	2.401 (2.129 - 2.709)	6.40E-44	gaba-derivative skeletal muscle relaxant
metoprolol tartrate	8636	1.546 (1.455 - 1.642)	1.97E-43	beta-adrenergic blocking agents
atorvastatin calcium	23854	1.339 (1.282 - 1.399)	3.43E-37	hmg-coa reductase inhibitors
levofloxacin	2936	1.800 (1.639 - 1.976)	7.15E-33	quinolone antibiotics
pantoprazole sodium	8029	1.482 (1.392 - 1.578)	1.44E-32	proton-pump inhibitors
buspirone hcl	1430	2.107 (1.858 - 2.390)	5.17E-29	anxiolytics sedatives and hypnotics misc
duloxetine hcl	3033	1.703 (1.551 - 1.870)	6.45E-27	sel.serotonin norepi reuptake inhibitor
fluticasone/vilanterol	1186	2.172 (1.895 - 2.491)	1.27E-26	orally inhaled preparations (steroids)
oxycodone hcl	1951	1.820 (1.626 - 2.038)	3.07E-23	opiate agonists
lisinopril/hydrochlorothiazide	2776	0.435 (0.371 - 0.511)	2.65E-22	angiotensin-converting enzyme inhibitors

hydralazine hcl	3364	1.585 (1.448 - 1.736)	3.17E-21	direct vasodilators
tiotropium bromide	1571	1.873 (1.655 - 2.121)	4.73E-21	antimuscarinics /antispasmodics
clonazepam	1720	1.797 (1.593 - 2.027)	1.55E-19	benzodiazepines (anticonvulsants)
insulin detemir	1524	1.839 (1.620 - 2.087)	5.17E-19	long-acting insulins
ranitidine hcl	2969	1.586 (1.441 - 1.746)	6.60E-19	histamine h2-antagonists
sulfamethoxazole/trimethoprim	3866	1.486 (1.363 - 1.621)	4.01E-17	sulfonamide antibiotics (systemic)
citalopram hydrobromide	2593	1.596 (1.440 - 1.768)	4.18E-17	selective-serotonin reuptake inhibitors
clopidogrel bisulfate	6397	1.365 (1.272 - 1.465)	7.24E-16	platelet-aggregation inhibitors
pregabalin	1568	1.737 (1.530 - 1.972)	1.81E-15	anticonvulsants misc
amoxicillin/potassium clav	4964	1.403 (1.297 - 1.517)	3.73E-15	aminopenicillin antibiotics
clotrimazole/betamethasone dip	1331	1.766 (1.540 - 2.025)	4.11E-14	azoles (skin and mucous membrane)
rosuvastatin calcium	6016	0.693 (0.633 - 0.759)	2.78E-13	hmg-coa reductase inhibitors
hydrochlorothiazide	8660	0.741 (0.688 - 0.798)	3.37E-13	thiazide diuretics
apixaban	4990	1.371 (1.267 - 1.484)	5.09E-13	direct factor xa inhibitors
rivaroxaban	2353	1.537 (1.379 - 1.713)	8.31E-13	direct factor xa inhibitors
tramadol hcl	6383	1.326 (1.235 - 1.425)	9.08E-13	opiate agonists
prednisone	8173	1.288 (1.207 - 1.374)	1.76E-12	adrenals
ketoconazole	1662	1.608 (1.417 - 1.825)	1.78E-11	azoles (skin and mucous membrane)

oxybutynin chloride	2221	1.516 (1.356 - 1.696)	3.17E-11	antimuscarinics
fluticasone propion/salmeterol	1894	1.557 (1.381 - 1.755)	4.46E-11	orally inhaled preparations (steroids)
mupirocin	2285	1.488 (1.331 - 1.662)	2.36E-10	antibacterials (skin mucous membrane)
doxycycline hyclate	4074	1.354 (1.241 - 1.477)	8.54E-10	tetracycline antibiotics
cephalexin	5444	1.299 (1.203 - 1.403)	2.84E-09	1st generation cephalosporin antibiotics
losartan/hydrochlorothiazide	2646	0.619 (0.538 - 0.714)	3.17E-09	angiotensin ii receptor antagonists
isosorbide mononitrate	2541	1.423 (1.279 - 1.584)	9.85E-09	nitrates and nitrites
fluticasone propionate	6686	1.259 (1.173 - 1.351)	1.95E-08	corticosteroids (eent)
dulaglutide	1150	1.622 (1.395 - 1.886)	2.97E-08	incretin mimetics
finasteride	2939	1.377 (1.245 - 1.523)	4.79E-08	5-alpha-reductase inhibitors
albuterol sulfate	7715	1.234 (1.154 - 1.319)	7.64E-08	selective beta-2-adrenergic agonists
torsemide	1420	1.528 (1.330 - 1.755)	1.78E-07	loop diuretics
methylprednisolone	3271	0.688 (0.609 - 0.778)	1.89E-07	adrenals
triamcinolone acetonide	3562	1.328 (1.210 - 1.458)	2.12E-07	corticosteroids (skin mucous membrane)
mirabegron	1209	1.561 (1.345 - 1.811)	3.93E-07	selective beta-3-adrenergic agonists
ciprofloxacin hcl	4594	1.282 (1.179 - 1.394)	4.88E-07	quinolone antibiotics
brimonidine tartrate	1272	1.534 (1.326 - 1.775)	7.72E-07	alpha-adrenergic agonists (eent)
chlorhexidine gluconate	1232	1.534 (1.322 - 1.779)	1.30E-06	eent anti-infectives misc

triamterene/hydrochlor othiazid	1529	0.583 (0.482 - 0.706)	2.40E-06	potassium-sparing diuretics
amoxicillin	5404	0.781 (0.712 - 0.856)	9.24E-06	aminopenicillin antibiotics
ezetimibe	1759	0.643 (0.542 - 0.763)	3.26E-05	cholesterol absorption inhibitors
bumetanide	1088	1.486 (1.267 - 1.742)	8.62E-05	loop diuretics
levothyroxine sodium	15117	1.137 (1.079 - 1.198)	0.000102 39	thyroid agents
amlodipine besylate	20300	1.121 (1.069 - 1.175)	0.000152 59	dihydropyridine s
budesonide/formoterol fumarate	1824	1.358 (1.196 - 1.543)	0.000184 46	orally inhaled preparations (steroids)
fluconazole	2113	1.331 (1.181 - 1.500)	0.000197 57	azole antifungals
metformin hcl	15386	0.879 (0.832 - 0.928)	0.000301 47	biguanides
clonidine hcl	1936	1.339 (1.182 - 1.516)	0.000308 44	central alpha-agonists
olmesartan medoxomil	1100	0.597 (0.478 - 0.745)	0.000377 38	angiotensin ii receptor antagonists
venlafaxine hcl	1324	1.405 (1.212 - 1.628)	0.000430 93	sel.serotonin norepi reuptake inhibitor
meloxicam	4355	0.794 (0.718 - 0.879)	0.000538 04	other nonsteroidal anti-inflam. agents
hydroxyzine hcl	1080	1.440 (1.226 - 1.692)	0.000648 36	anxiolytics sedatives and hypnotics misc
losartan potassium	12578	0.876 (0.825 - 0.930)	0.000925 54	angiotensin ii receptor antagonists
nitrofurantoin monohyd/m-cryst	2665	1.272 (1.141 - 1.417)	0.000941 01	urinary anti-infectives
lovastatin	1615	0.681 (0.572 - 0.810)	0.000987 71	hmg-coa reductase inhibitors
latanoprost	4456	1.210 (1.110 - 1.319)	0.001028 92	prostaglandin analogs

sitagliptin phosphate	2331	1.288 (1.148 - 1.445)	0.00105144	dipeptidyl peptidase-4(dpp-4) inhibitors
allopurinol	4642	1.204 (1.106 - 1.311)	0.00112362	antigout agents
oxycodone hcl/acetaminophen	2733	1.248 (1.121 - 1.391)	0.00337478	opiate agonists
pravastatin sodium	5583	0.846 (0.775 - 0.923)	0.0107583	hmg-coa reductase inhibitors
ipratropium bromide	1071	1.357 (1.151 - 1.601)	0.01734722	eent drugs misc
simvastatin	8965	0.880 (0.821 - 0.943)	0.01774904	hmg-coa reductase inhibitors
hydrocortisone	1339	1.316 (1.133 - 1.528)	0.01855794	corticosteroids (skin mucous membrane)
empagliflozin	1043	0.674 (0.542 - 0.837)	0.02065085	sodium-gluc cotransport 2 (sglt2) inhib
colchicine	1099	1.335 (1.133 - 1.572)	0.03157155	antigout agents
ropinirole hcl	1019	1.339 (1.130 - 1.588)	0.0415578	nonergot-deriv.dopamine receptor agonist
lisinopril	14848	1.095 (1.038 - 1.154)	0.04227668	angiotensin-converting enzyme inhibitors
chlorthalidone	1281	0.724 (0.598 - 0.876)	0.04634958	thiazide-like diuretics
