# Supplementary material for: Membrane-wide screening identifies potential tissue-specific determinants of SARS-CoV-2 tropism
Source: PLoS Pathog. 2025 Jul 17;21(7):e1013157. doi: 10.1371/journal.ppat.1013157 (PMC12286382; doi:10.1371/journal.ppat.1013157)
Supplement: S3 Table — (PDF) [file ppat.1013157.s003.pdf]

Supplementary Table 3.
List of cDNA plasmids generated for validation of CRISPRa SARS-CoV-2 screen top hits

Gene	Vector	Vendor	Vendor ID	Cloning Technique
BFP	pLenti	n/a	n/a	Gibson
ACE2	pDONR221	DNASU	HsCD00829706	Gateway
TMPRSS2	pLenti6.3/V5-DEST	DNASU	HsCD00939610	n/a
HLA-DRA	pLenti6.3/V5-DEST	DNASU	HsCD00943681	n/a
ANO5	pLenti6.3/V5-DEST	DNASU	HsCD00937305	n/a
SMPD2	pLenti6.3/V5-DEST	DNASU	HsCD00943698	n/a
TSPAN15	pLenti6.3/V5-DEST	DNASU	HsCD00938721	n/a
NETO1	pLenti6.3/V5-DEST	DNASU	HsCD00951550	n/a
MALL	pLenti6.3/V5-DEST	DNASU	HsCD00940976	n/a
OLFML3	pLenti6.3/V5-DEST	DNASU	HsCD00940741	n/a
LGMN	pDONR221	DNASU	HsCD00076382	Gateway
ADAM19	pLenti6.3/V5-DEST	DNASU	HsCD00951521	n/a
CYSLTR2	pLenti6.3/V5-DEST	DNASU	HsCD00936232	n/a
NRP2	pDONR223	DNASU	HsCD00398541	n/a
STX1B	pLenti6.3/V5-DEST	DNASU	HsCD00937676	n/a
SCNN1D	pLenti6.3/V5-DEST	DNASU	HsCD00870284	n/a
HLA-DOB	pLenti6.3/V5-DEST	DNASU	HsCD00939113	n/a
IL10RB	pLenti6.3/V5-DEST	DNASU	HsCD00943063	n/a
CNTN6	pLenti6.3/V5-DEST	DNASU	HsCD00866637	n/a
ZBTB11	pLenti6.3/V5-DEST	DNASU	HsCD00866799	n/a
EXT2	pLX304	GeneCopoeia	EX-OL00524-LX304-B	n/a

HLA-DPB1	pLenti6.3/V5-DEST	DNASU	HsCD00939016	n/a
LRRC8D	pDONR221	DNASU	HsCD00828954	Gateway
STOM	pLVr105	GeneCopoeia	EX-Z7517-Lv105	n/a
ADAM2	pLX304	GeneCopoeia	EX-OL10829-LX304	n/a
ISLR	pLenti6.3/V5-DEST	DNASU	HsCD00953940	n/a
SLC6A4	pShuttle	GeneCopoeia	GC-A0200	Gateway
SLC26A9	pDONR221	Addgene	132140	Gateway
KCND1	pLenti6.3/V5-DEST	DNASU	HsCD00939807	n/a
STX18	pLenti6.3/V5-DEST	DNASU	HsCD00938917	n/a
ADAM9	pShuttle	GeneCopoeia	GC-X1023-CF	Gateway
KCNA6	pLX304	DNASU	HsCD00439521	n/a
CD7	pLenti6.3/V5-DEST	DNASU	HsCD00943071	n/a
IL7R	pLenti6.3/V5-DEST	DNASU	HsCD00939421	n/a
GPR108	pLenti6.3/V5-DEST	DNASU	HsCD00830792	n/a
ICMT	pLenti6.3/V5-DEST	DNASU	HsCD00942999	n/a
TMEM151A	pDONR221	DNASU	HsCD00963937	Gateway
LSR	pENTR223	DNASU	HsCD00516191	Gateway
ACE	pDONR221	DNASU	HsCD00821132	Gateway
RDH10	pLenti6.3/V5-DEST	DNASU	HsCD00831751	n/a
EPHA4	pLenti6.3/V5-DEST	DNASU	HsCD00860721	n/a
