# Supplementary figures and images for: Membrane-wide screening identifies potential tissue-specific determinants of SARS-CoV-2 tropism
Source: PLoS Pathog. 2025 Jul 17;21(7):e1013157. doi: 10.1371/journal.ppat.1013157 (PMC12286382; doi:10.1371/journal.ppat.1013157)

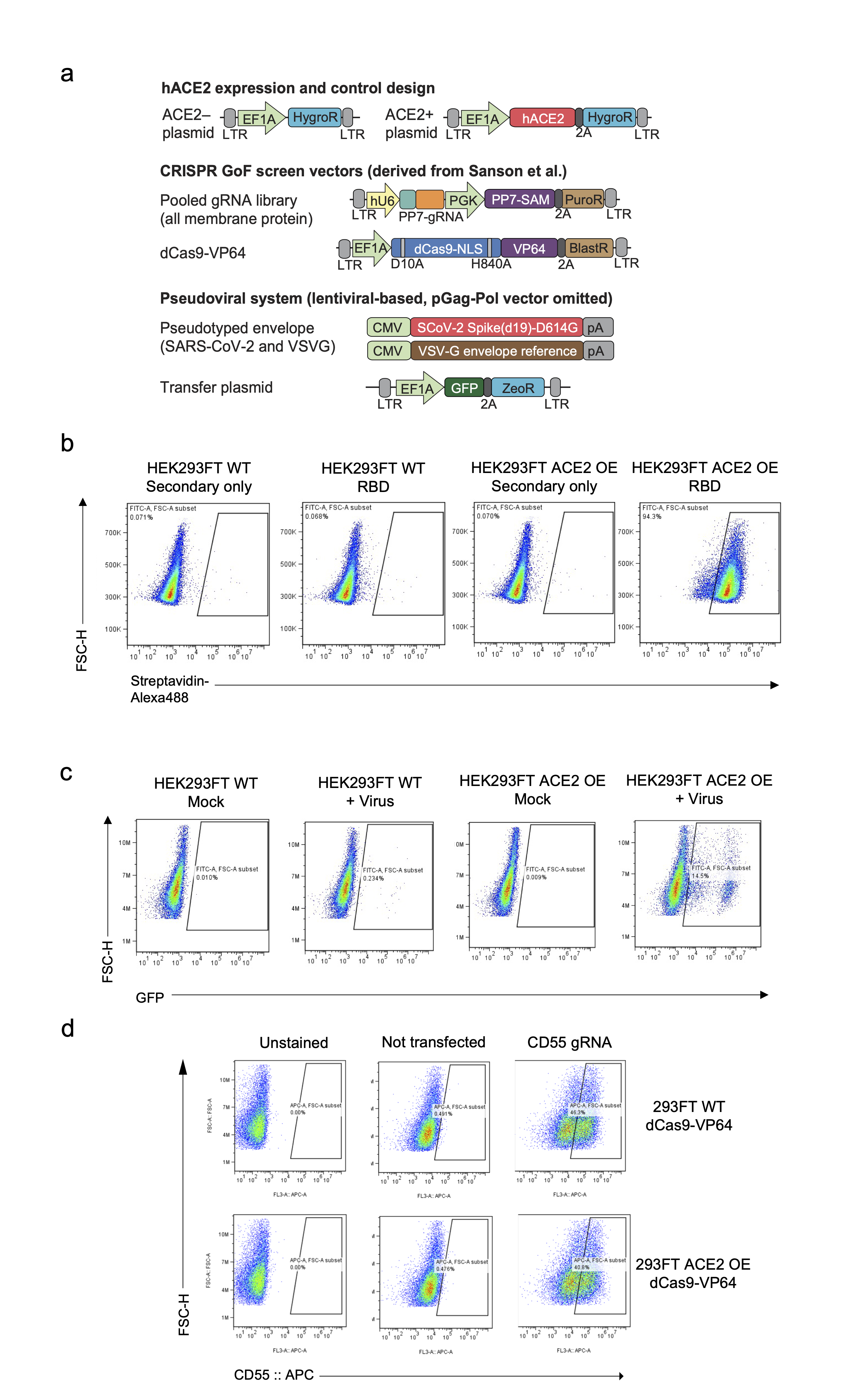

Supplement: S1 Fig — a, Schematics showing the design of vector systems used in CRISPRa screening. b, WT and ACE2 OE lines stained with or without RBD-Biotin and Streptavidin-Alexa488. c, WT and ACE2 OE cells either mock infected or infected with SARS-CoV-2 D614G Spike pseudotyped lentiviruses. d,Transfection of 293FT WT and ACE2 OE lines expressing dCas9-VP64 with pXPR_502 vector containing a CD55 targeting gRNA. (TIFF) [file ppat.1013157.s008.tiff]

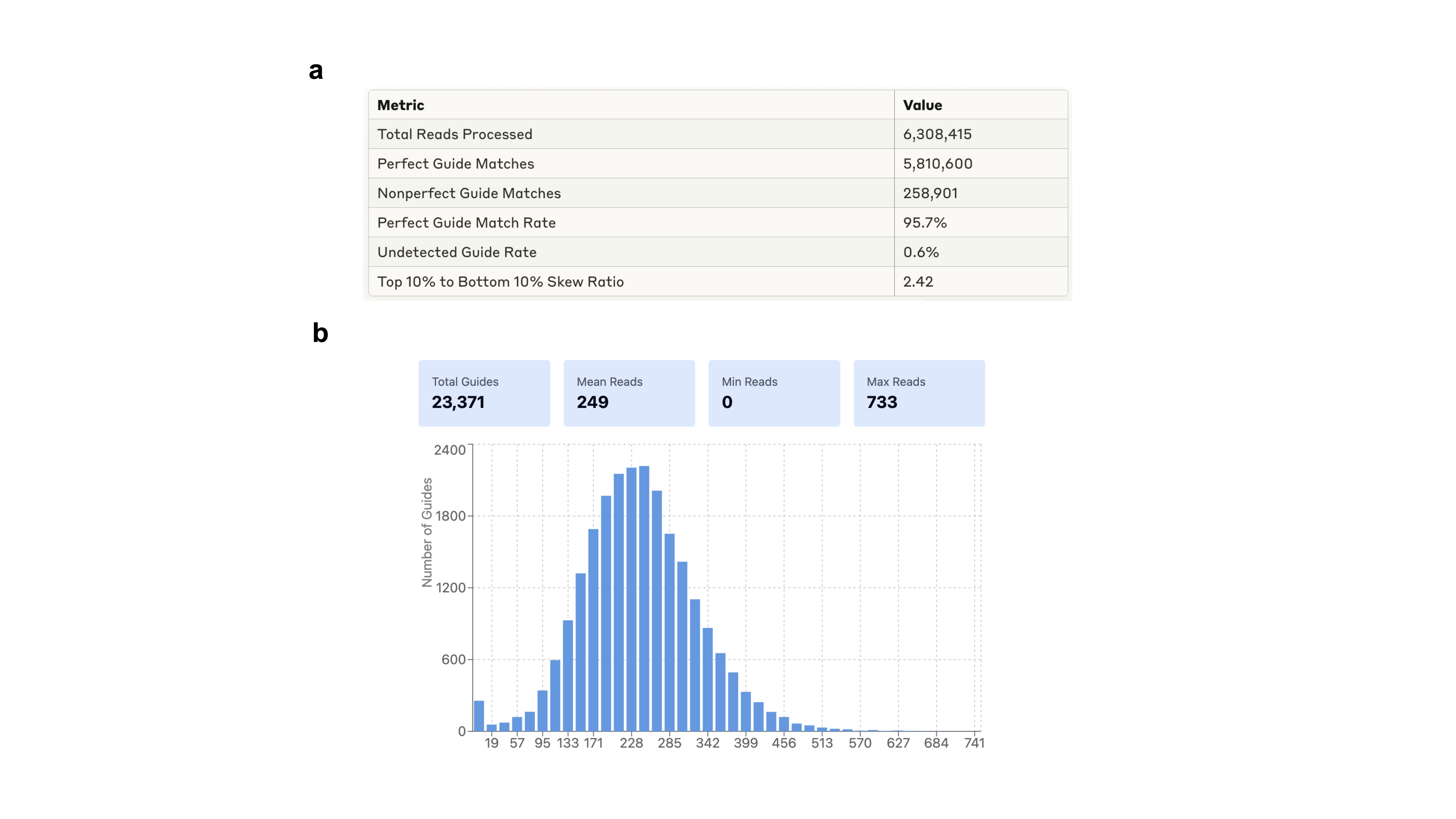

Supplement: S2 Fig — a, Statistics for sequencing of membrane-wide CRISPRa plasmid library. b, Distribution of gRNAs and representation in the membrane-wide CRISPRa plasmid library. (TIFF) [file ppat.1013157.s009.tiff]

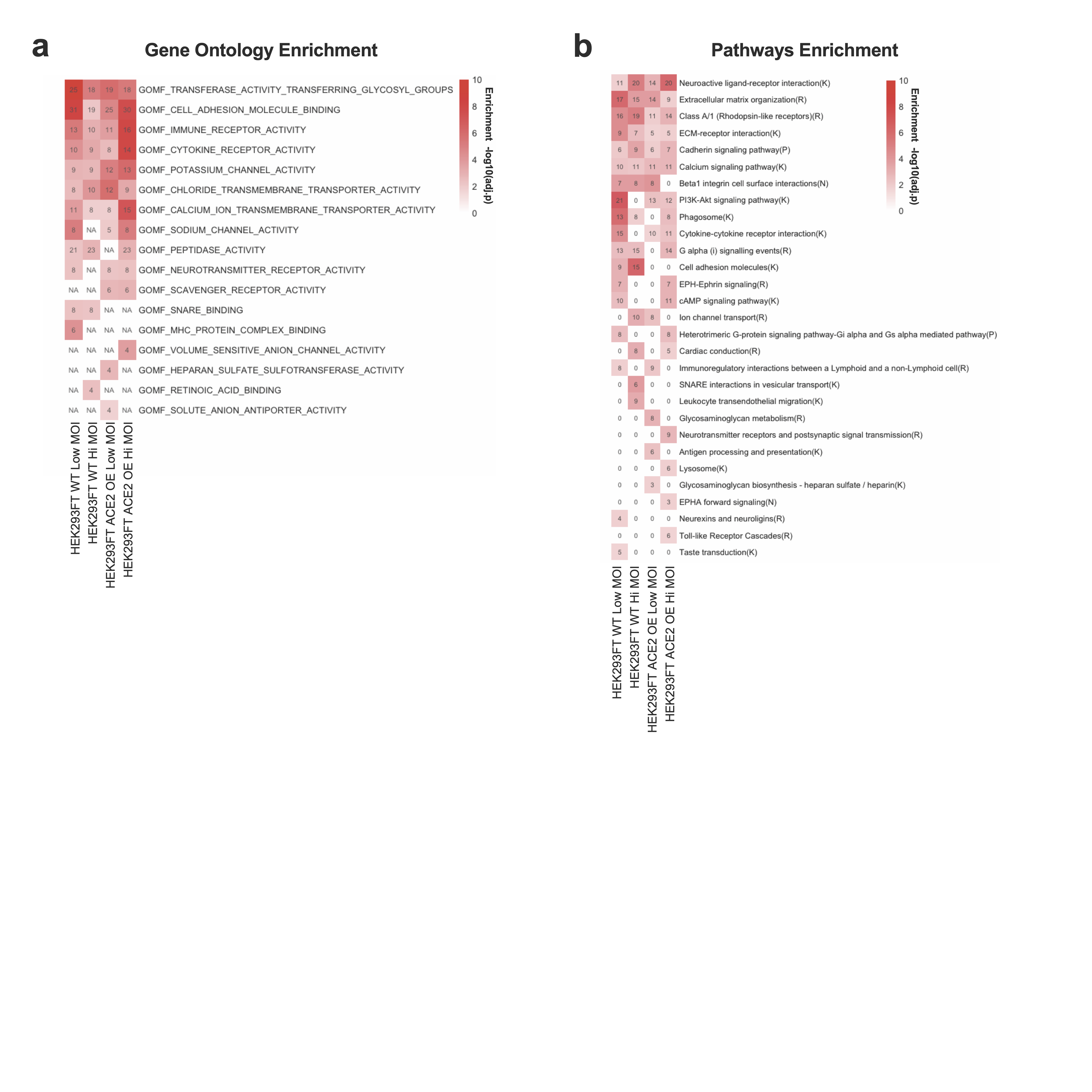

Supplement: S4 Fig — Heatmap showing the overall human tissue expression patterns of top-ranking genes using GTEX v8 dataset. (TIFF) [file ppat.1013157.s011.tiff]

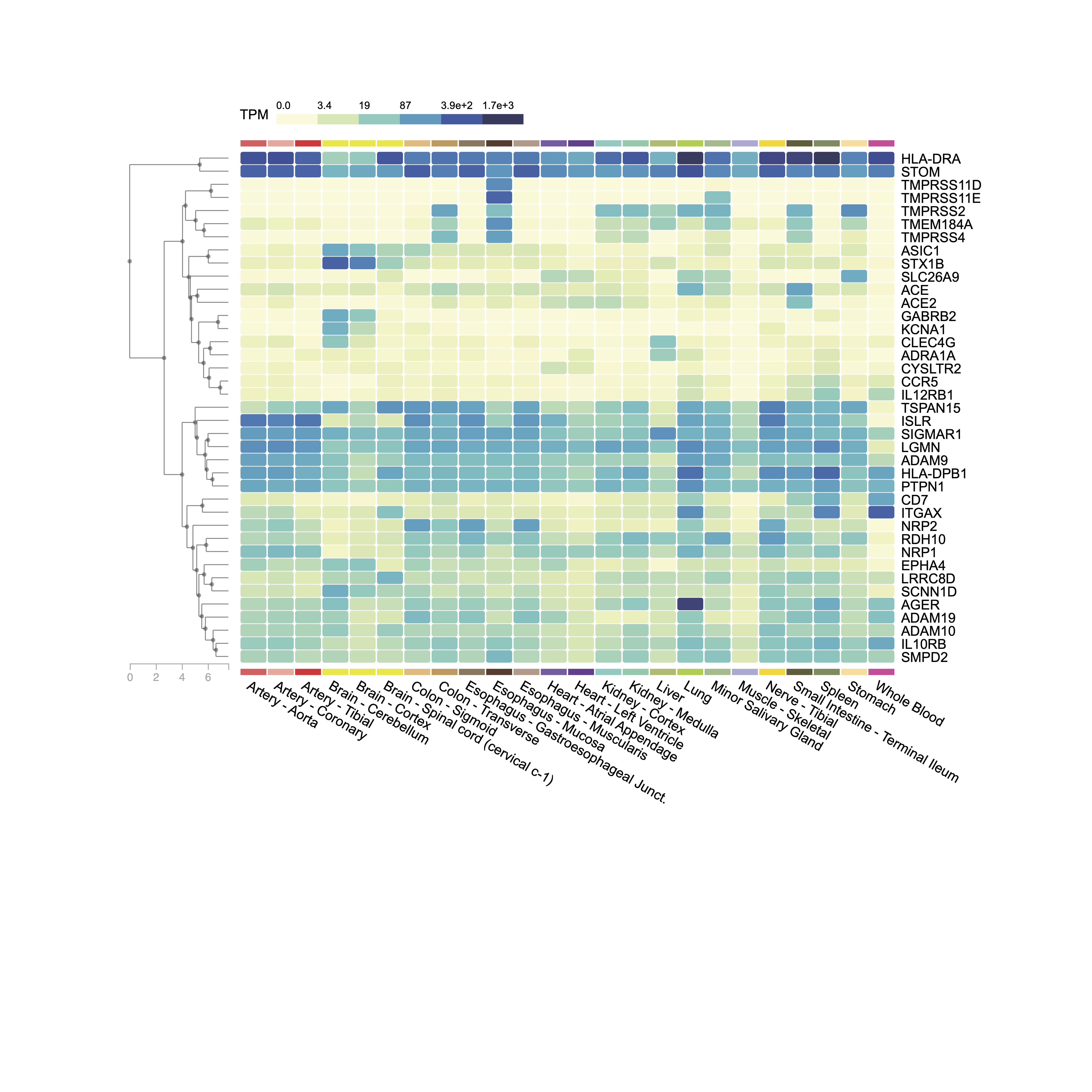

Supplement: S5 Fig — a-b, Gene set overlap analysis using (a) gene ontology (GO) (b) pathways on top 10% of hits from each screen condition. The top GO terms or pathways of each screen condition were selected for visualization. (TIFF) [file ppat.1013157.s012.tiff]

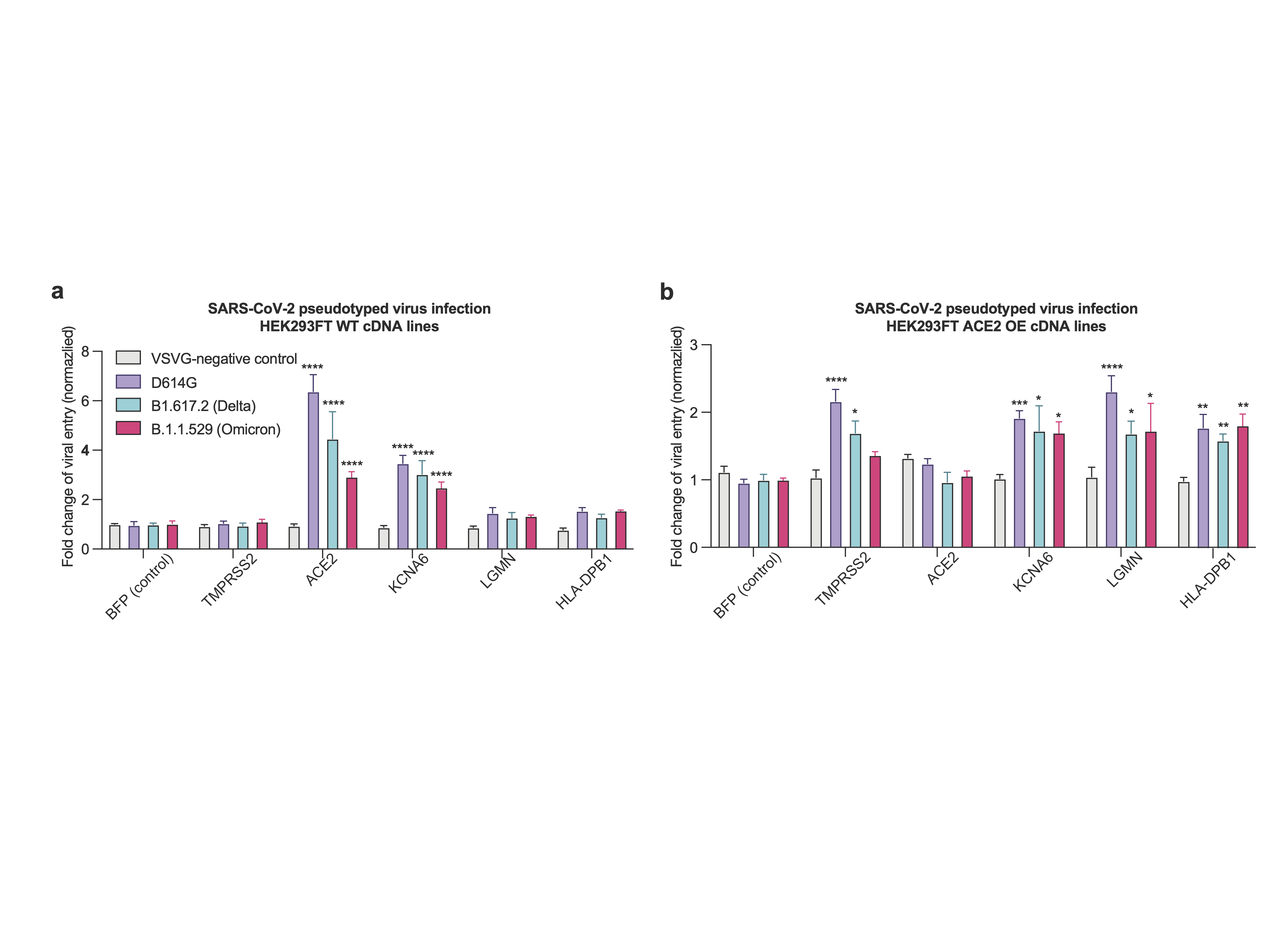

Supplement: S6 Fig — (a-b) (a) HEK293FT WT lines and (b) ACE2 OE HEK293FT lines stably overexpressing cDNAs of putative stably overexpressing cDNAs of putative SARS-CoV-2 entry factors were transduced with lentiviruses pseudotyped with either VSVG, Spike D614G variant, Spike B1.617.2 (Delta), Spike B.1.1.529 (Omicron) variant. (TIFF) [file ppat.1013157.s013.tiff]

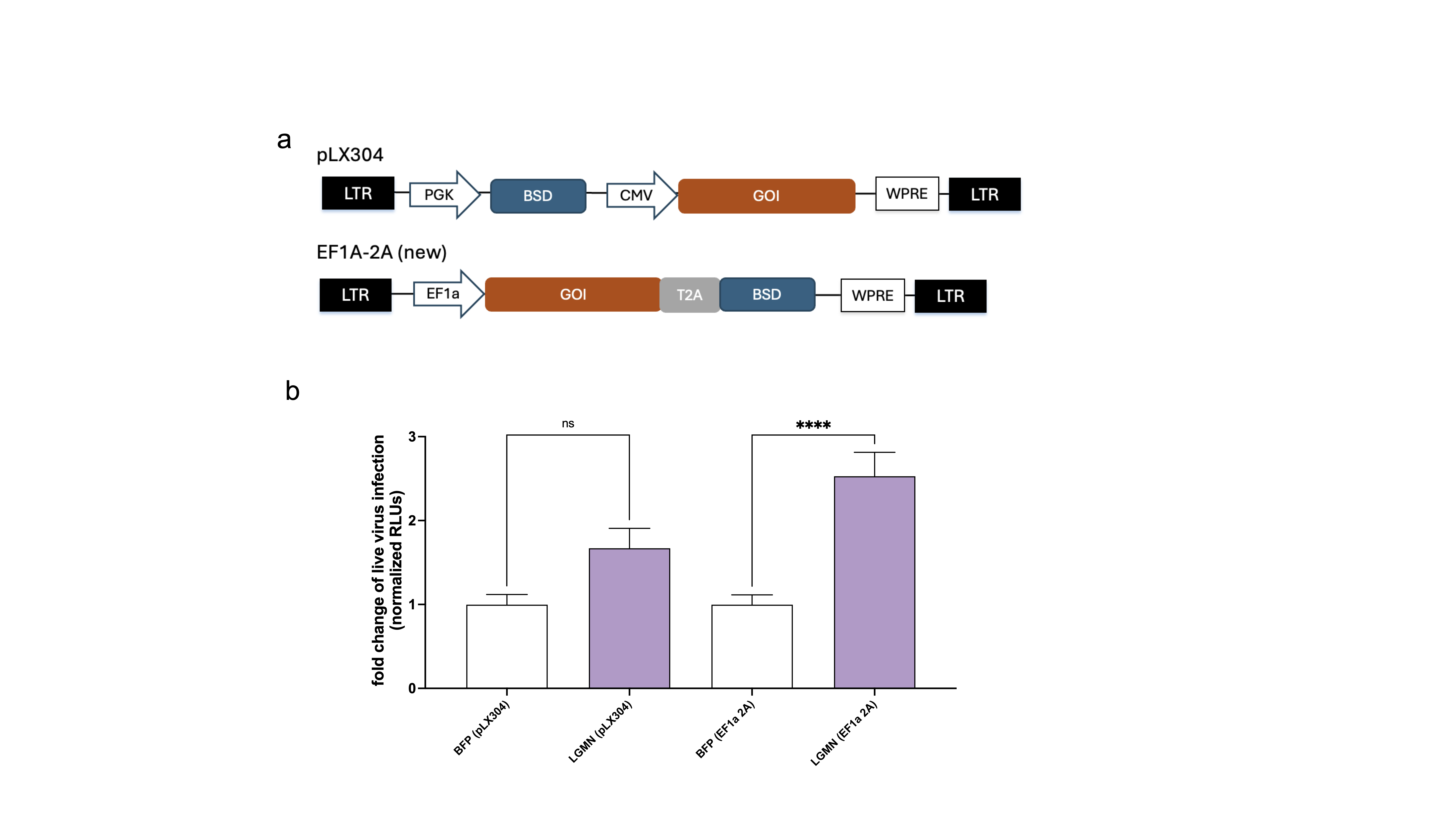

Supplement: S8 Fig — a, Design of pLX304 and EF1a-2A vectors. LTR: Long Terminal Repeat, PGK: human phosphogylcerate kinase promoter, BSD: blasticidin deaminase, CMV: cytomegalovirus promoter, GOI: gene of interest, WPRE: woodchuck hepatitis virus postregulatory element, P2A: porcine teschovirus-1 2A peptide. b, Replication competent SARS-CoV-2 infection of 293FT ACE2 OE cells expressing BFP or LGMN cloned into pLX304 or EF1a-2A. Two independent experiments, six technical replicates per experiment. Statistical analyses: two-way ANOVA with correction for multiple comparisons during hypothesis testing. *, p < 0.05; **, p < 0.01; ***, p < 0.001; ****, p < 0.0001. (TIFF) [file ppat.1013157.s015.tiff]

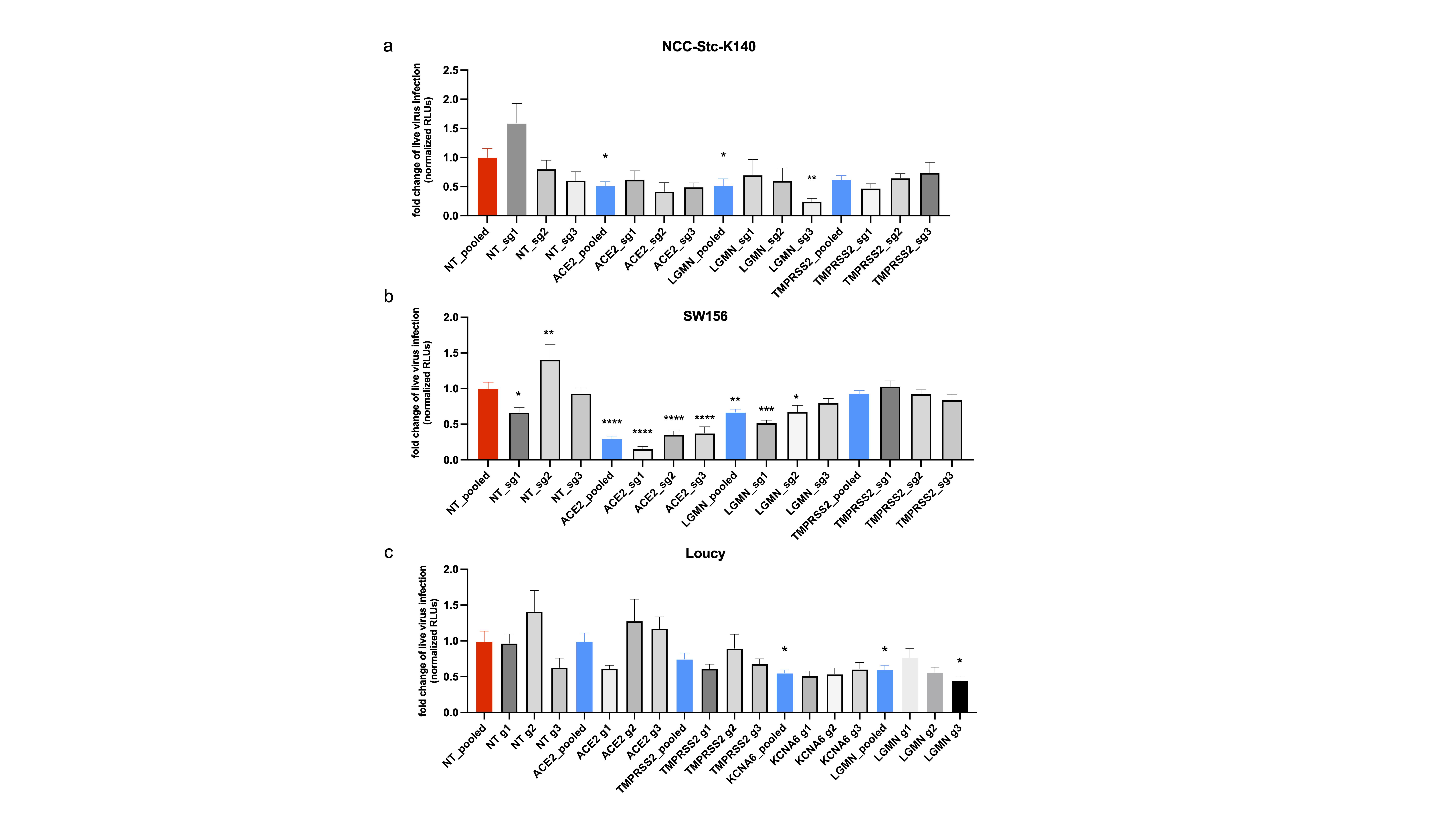

Supplement: S9 Fig — SARS-CoV-2 live virus infection of NCC-Stc-K140 (a) SW156 (b) and Loucy (c) cells perturbed with CRISPR-based loss-of-function constructs. Three guideRNAs were used per gene and results are reported for individual guide RNAs and pooled groups for analysis. Infections was repeated twice to collect replicates for all cell lines. All data represent mean with SEM. Statistical analyses: one-way ANOVA with non-target (NT) pooled as the control condition with correction for multiple comparisons during hypothesis testing. Two independent experiments, 3–6 technical replicates per experiment. *, p < 0.05; **, p < 0.01; ***, p < 0.001; ****, p < 0.0001. (TIFF) [file ppat.1013157.s016.tiff]

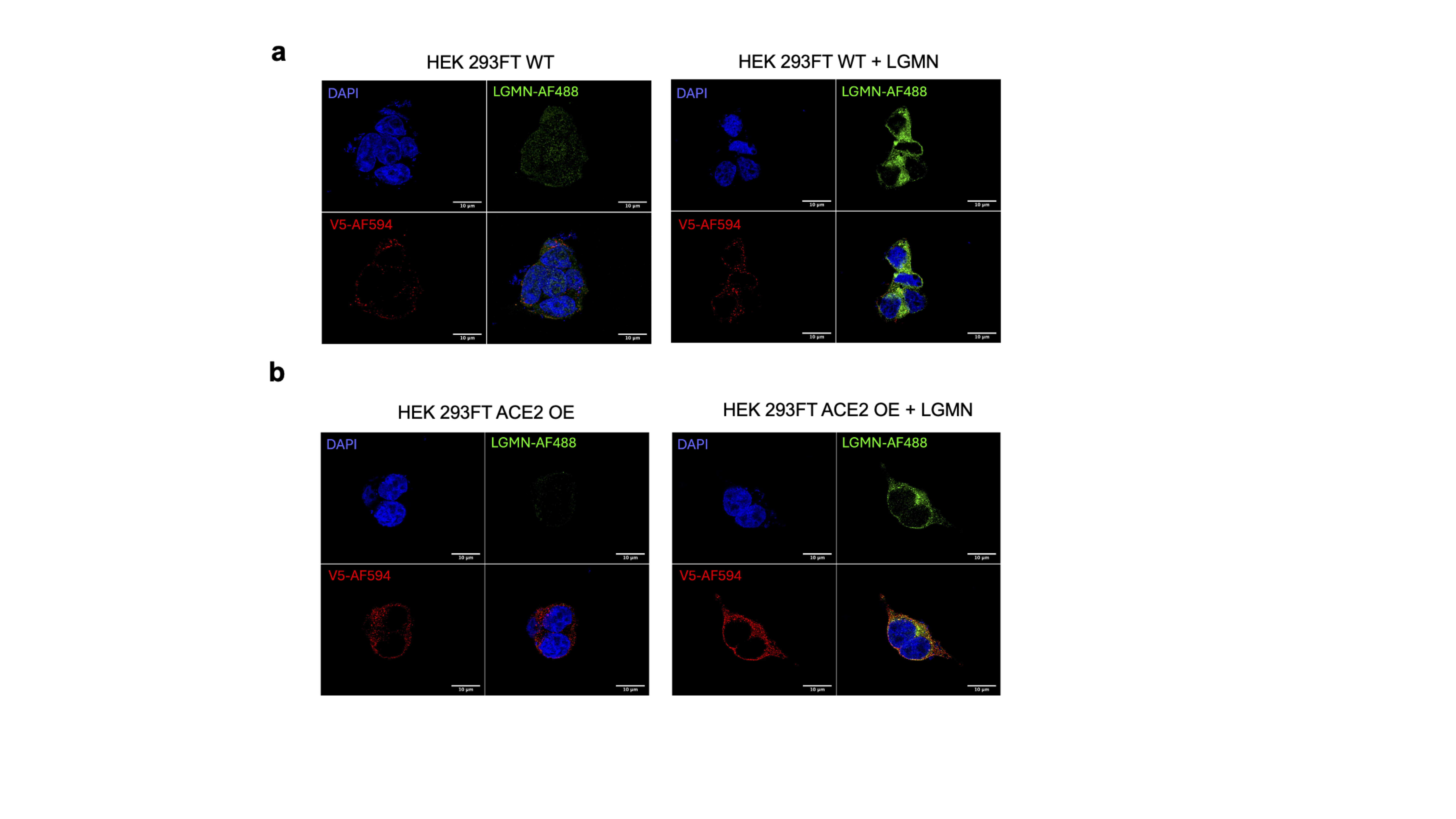

Supplement: S10 Fig — a-b, 293FT WT (a) or ACE2 OE (b) cells with or without exogenous LGMN expression were transduced with Spike-V5tag pseudotyped lentivirus, then stained by immunofluorescence assay imaged with a confocal microscope at 4 hr post-transduction. Blue: DAPI; green: LGMN protein; red: Spike-V5 protein. Scale Bar: 10 µm. (TIFF) [file ppat.1013157.s017.tiff]

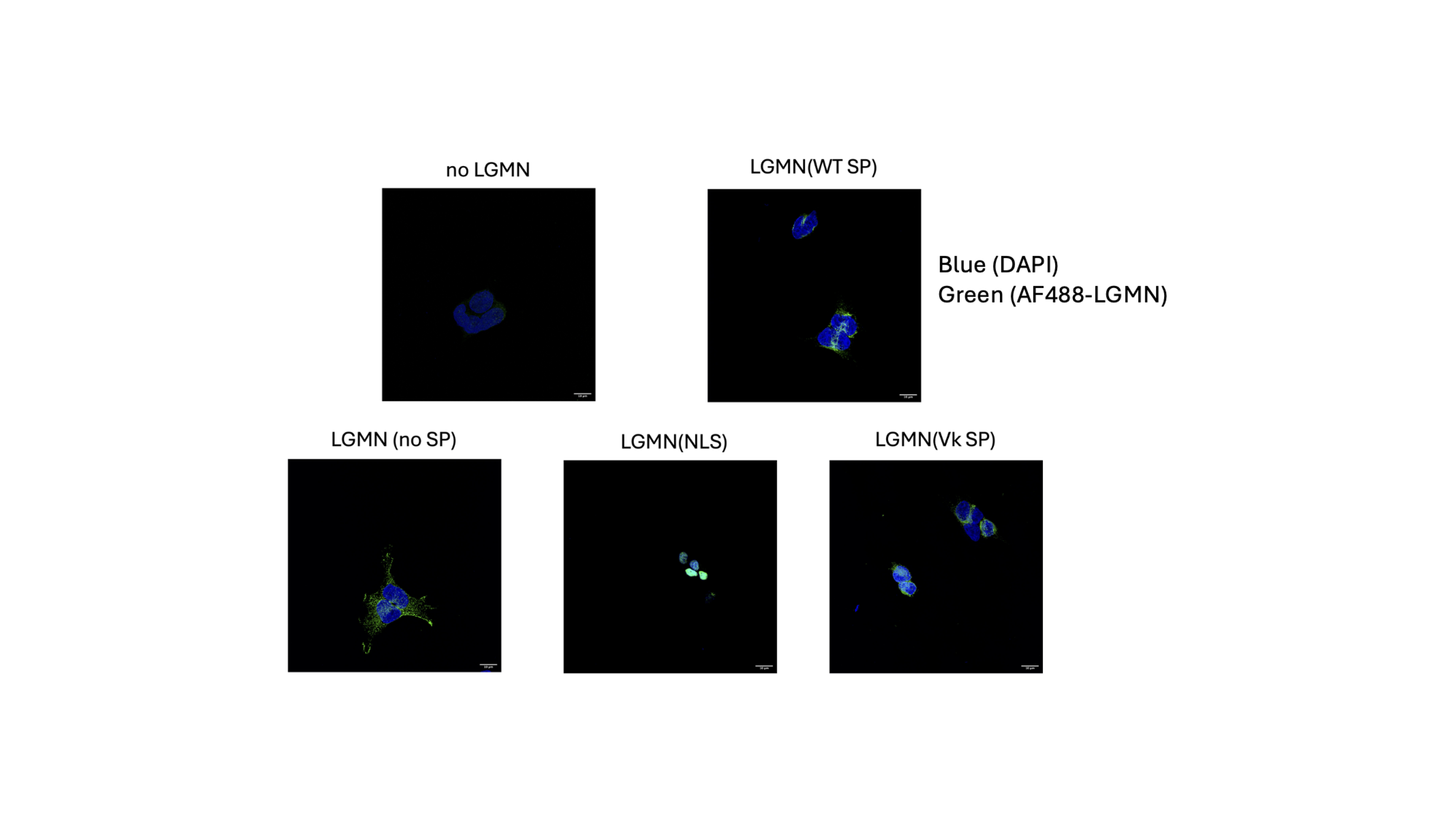

Supplement: S11 Fig — 293FT ACE2 OE cells transduced with LGMN or LGMN signal peptide variants were stained by immunofluorescence assay and imaged with a confocal microscope. Blue: DAPI, green: LGMN protein. Scale Bar: 10 µm. (TIFF) [file ppat.1013157.s018.tiff]

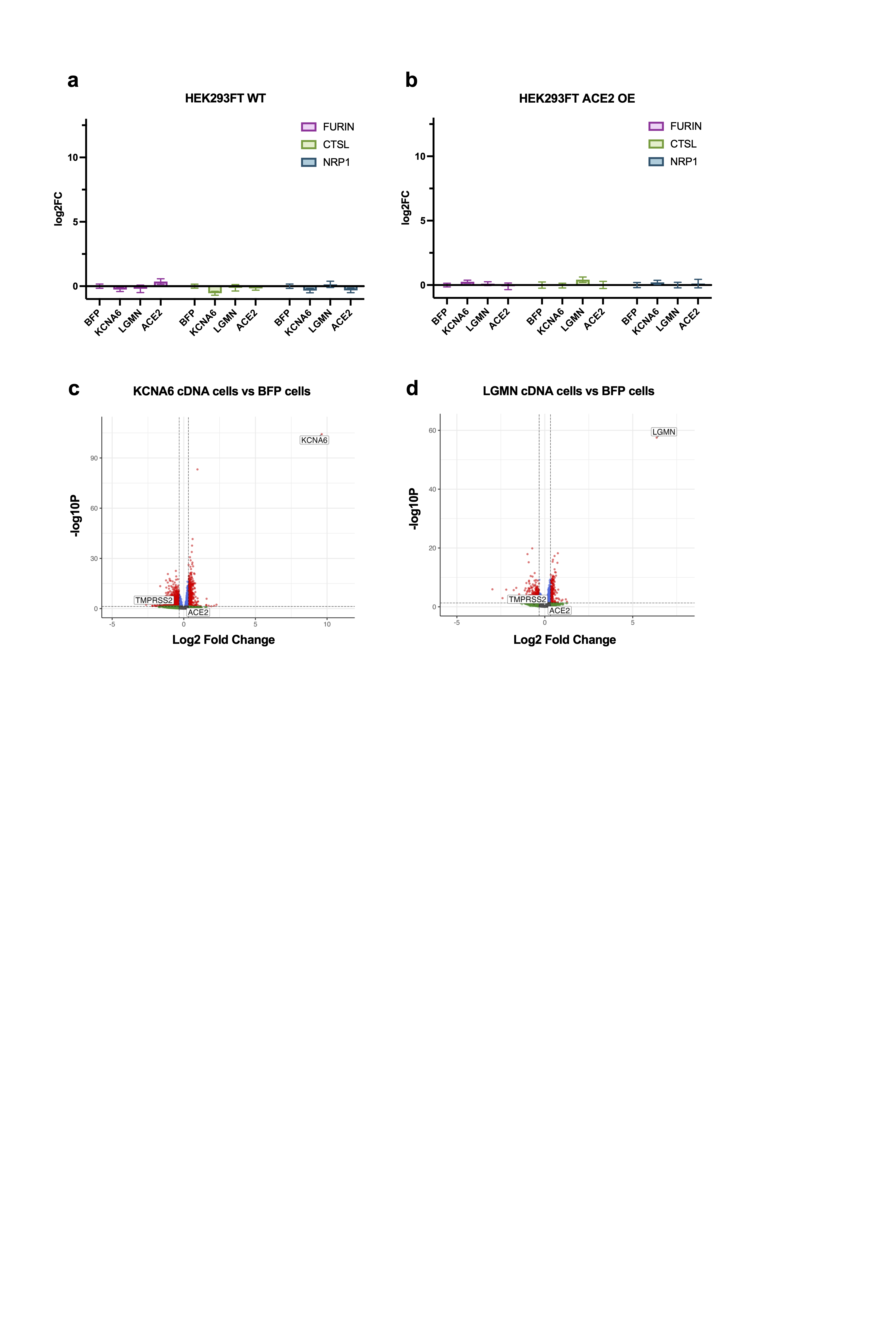

Supplement: S12 Fig — a, KCNA6 genome annotations in the GRCh38 (hg38) and GRCh37 (hg19) references using NCBI Genome Data Viewer. (b) Expression of ACE2 and KCNA6 in the single-cell RNA-seq data of olfactory neuroepithelium using different versions of genome references (from Durante et al.) [51]. Cell Ranger 6.0 was used for all alignments and the expression was calculated by averaging the ACE2/KCNA6 expression in all cells and normalized to the ACE2 expression from the standard GRCh38 reference genome. (c) Average expression of ACE2/KCNA6 in olfactory epithelium using the Salmon - Alevin pipeline, calculated similarly as in panel C. The standard GRCh37/GRCh38 genome references were used. (d) UMAP depicting the olfactory epithelial cell types from two patients. The cell cluster identities were based on Durante et al. [51] (e) UMAPs depicting the expression levels of KCNA6 in individual patients. (f) Focused UMAPs of the neuronal populations showing co-expression of KCNA6 and OLIG2, a reported marker for virus-infected neuronal cells in COVID19 patient olfactory neuroepithelium. (TIFF) [file ppat.1013157.s019.tiff]

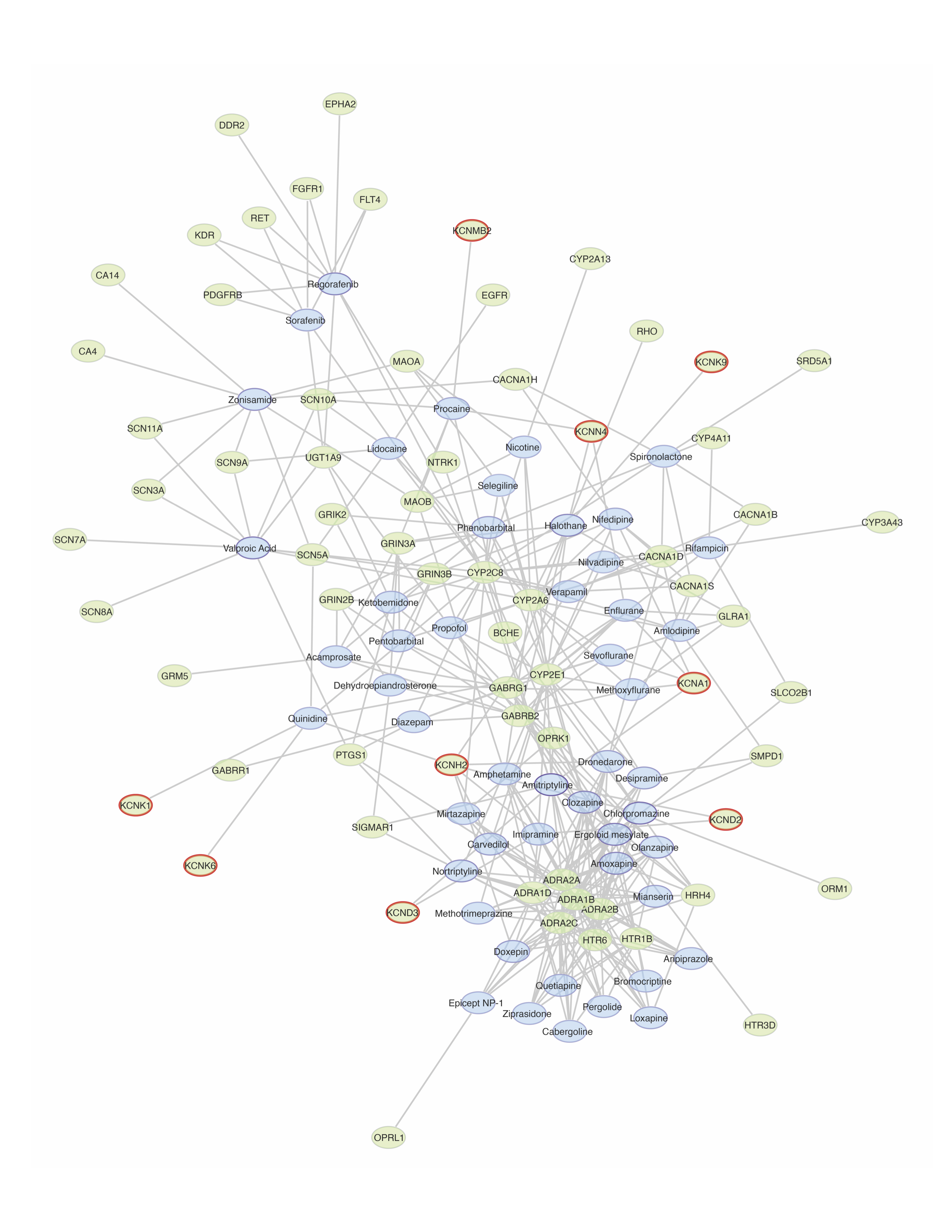

Supplement: S14 Fig — Overview of the drug-target interaction network, showing an induced subgraph of the 50 highest ranked compounds (drugs in blue; screen hits in green; potassium channel genes outlined in red). (TIFF) [file ppat.1013157.s021.tiff]

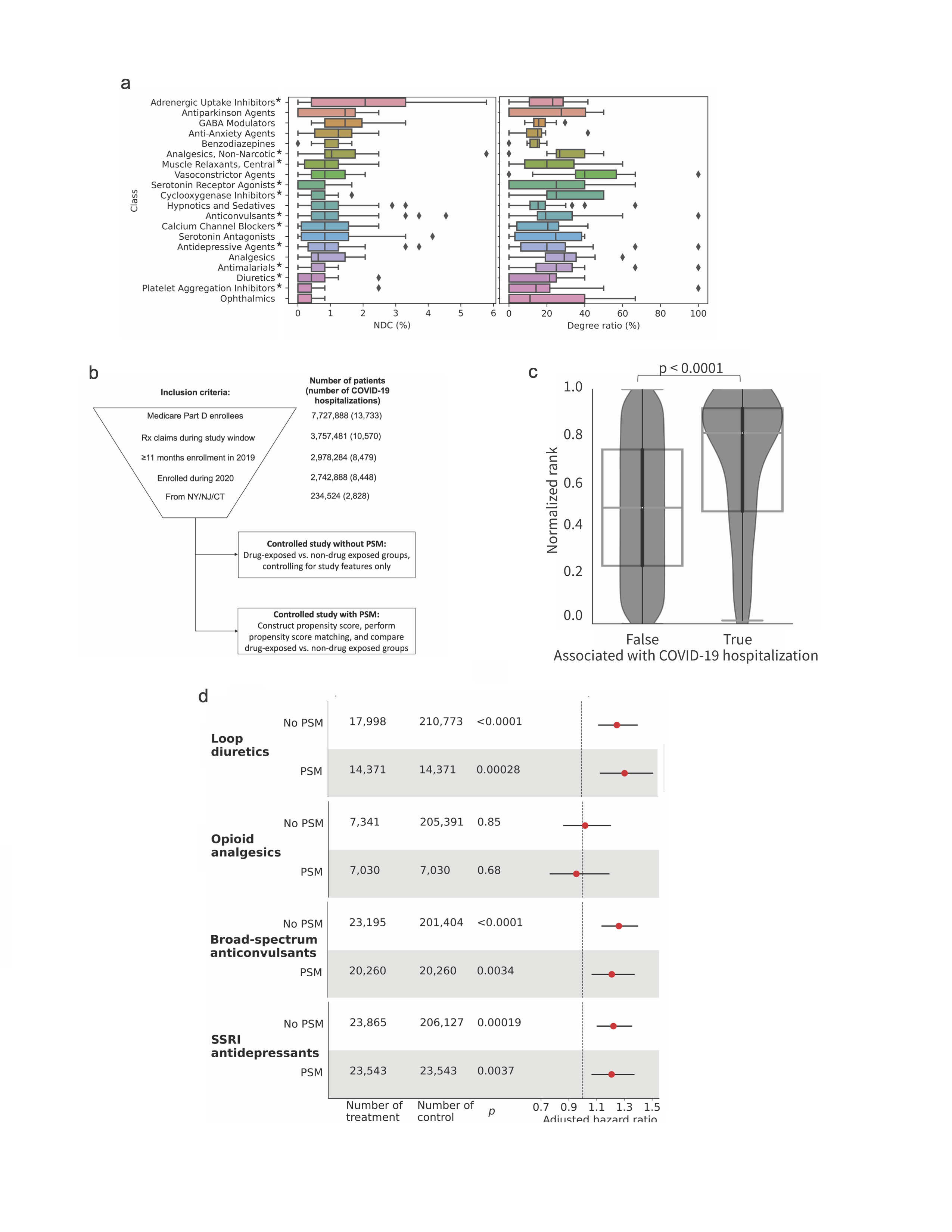

Supplement: S15 Fig — a, Top drug classes enriched in hits from the interaction network model by NDC and degree ratio with respect to screen hits. Asterisks indicate drug classes with at least one member targeting a potassium channel. b, Controlled study design for COVID-19 hospitalization from pharmaceutical claims data. c, Association between COVID-19 hospitalization in the unmatched study and drugs ranked highly in the drug-target interaction network. d, Real world evidence for associations between ion-channel-targeting drug classes identified in the screen and increased risk of COVID-19 hospitalization in propensity-score-matched subjects. (TIFF) [file ppat.1013157.s022.tiff]
