# Supplementary material for: Membrane-wide screening identifies potential tissue-specific determinants of SARS-CoV-2 tropism
Source: PLoS Pathog. 2025 Jul 17;21(7):e1013157. doi: 10.1371/journal.ppat.1013157 (PMC12286382; doi:10.1371/journal.ppat.1013157)
Supplement: Attachment — Submitted filename: PLOSPathogens Response final.pdf [file ppat.1013157.s024.pdf]

Response to Reviewers' Comments:

RE: We thank the reviewers for their positive comments and helpful suggestions on our manuscript, we have substantially revised our manuscript with new data, revised figures/texts according to the reviewers' suggestions, and enhanced the clarity of presentations thanks to the comments.

Specifically, our revisions include:

- New and repeated experiments using a freshly generated LGMN cDNA line to reconcile differences between pseudovirus and live virus infection data.
- A tempered interpretation of LGMN's mechanism of action, along with a schematic of its domain organization.
- We are also including new data (in addition to the previous protease activity mutation study) to show that membrane targeting is critical for the LGMN effect on viral infection. This data is supported by confocal microscopy data showing altering the LGMN signal peptide alters localization in the cell.
- Detailed quality metrics for the CRISPRa libraries utilized.
- Expanded text to explain the rationale for candidate selection and an updated overlap analysis with recently published GOF screens.

We hope that these revisions satisfactorily address the reviewers' concerns and enhance the overall quality and clarity of our manuscript.

Reviewers' Comments:

Part I - Summary
Please use this section to discuss strengths/weaknesses of study, novelty/significance, general execution and scholarship.

Reviewer #1: In the work by Ravi K. Dinesh et al., membrane-wide CRISPR activation screen was used to identify novel SARS-CoV-2 host factors. Validation experiments with replication-competent SARS-CoV-2 confirmed the role of newly identified host factors, including endo-lysosomal protease legumain (LGMN) and the potassium channel KCNA6. Using clinical data, they find possible associations between expression of either LGMN or KCNA6 and SARS-CoV-2 infection in human tissues. Overall, the experimental results were original and informative. The concerns/suggestions are listed below:

RE: We thank the reviewers for the positive comments.

Major:
1. In the introduction (Pg3, Line 30), the authors reviewed current therapeutics for SARS-CoV-2, including antibodies and small molecules. There are also investigational peptidyl therapeutics

targeting the fusion process (for example, Xia et al., 2021, Signal Transduct Target Ther). These therapeutics should be reviewed in a more comprehensive approach.

RE: We thank the reviewer for the suggestion regarding the review of peptidyl therapeutics targeting the fusion process. We have now added a discussion of investigational peptidyl therapeutics in the introduction of the revised manuscript (page 3, line 37-40).

2. Fig 2. According to the screen result in Fig 1g and pseudovirus result in Fig 2b, LGMN OE could promote virus infection in HEK293FT ACE2 OE cells. The results in Fig 2f showed that LGMN OE could not promote live virus infection. The author should provide an explanation to this, either in the results or discussion, as well as the differential effects of LGMN in WT and ACE2OE cell line (Fig 2e and 2f)

RE: We appreciate the reviewer's observation regarding the differential levels of significance observed between pseudovirus and live virus experiments in ACE2 OE 293FT in Figure 2. We had shown in our initial submission in our LGMN catalytic mutant experiment (**Figure 3f, currently Figure 3g**) that LGMN overexpression in ACE2 OE cells lead to statistically significant differences in replication-competent SARS-CoV-2 infection compared to the BFP control. We believe the discrepancy in the results is due to the difference in vectors used between these two sets of experiments. To confirm this we compared 293FT ACE2 OE cells transduced with both old (pLX304 backbone) and new (EF1a-2a backbone) vectors expressing LGMN and BFP. We show in **Supplementary Figure 8** that the newer vector showed more robust effects due to LGMN overexpression. We include a discussion of why we might observe these differences in the results section of the manuscript (**lines 29-41**).

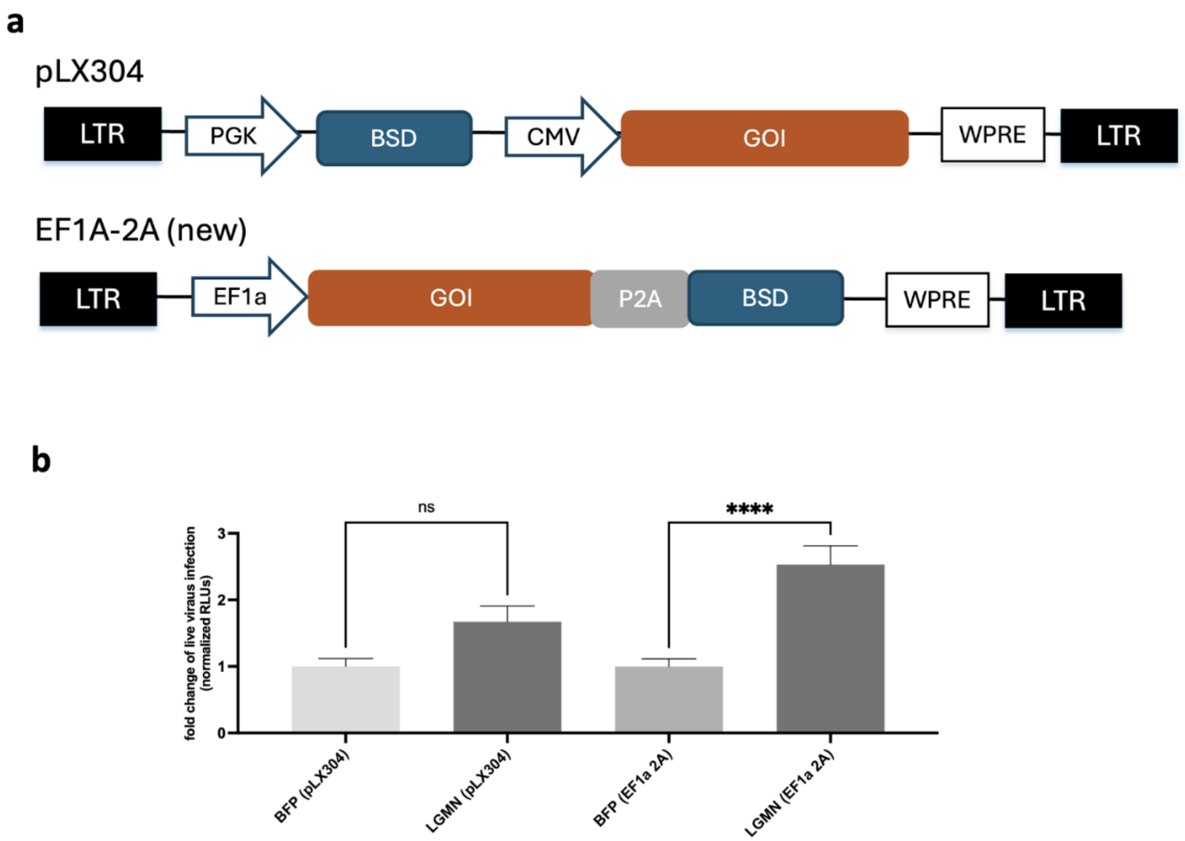

New Supplementary Fig. 8. Comparison of LGMN activity and expression with different expression vectors. a, Design of pLX304 and EF1a-2A vectors. LTR: Long Terminal Repeat, PGK: human phosphogylcerate kinase promoter, BSD: blasticidin deaminase, CMV: cytomegalovirus promoter, GOI: gene of interest, WPRE: woodchuck hepatitis virus postregulatory element, P2A: porcine teschovirus-1 2A peptide. b, Replication competent SARS-CoV-2 infection of 293FT ACE2 OE cells expressing BFP or LGMN cloned into pLX304 or EF1a-2A. The new vector has better expression and more robust activities.

3. Fig 3. The authors' conclusions on Fig. 3 are: 1) The function of LGMN is dependent on ACE2; 2) the function of LGMN is the same to TMPRSS2. However, none of the above conclusion/hypothesis is fully supported by the presented data. First, comparing the data in Fig.2e and 2f, one would find that the effects of LGMN on viral infection is more profound in the absence of ACE2 OE. This seemed to be opposite to what the authors claimed. To illustrate the dependency of the function of LGMN on ACE2, double knockout cell lines should be made and examined for viral infection (as shown in subsequent section). Second, in order to claim LGMN can replace the function of TMPRSS2, the authors would need to demonstrate that LGMN and TMPRSS2 can process/cleave spike protein in the same form at the same stage of viral infection. Given the subcellular localization of LGMN as the authors stated (in endosome and lysosome), LGMN is unlikely to cleave spike protein at the very early stage of infection to trigger the membrane fusion. Rather, LGMN is more likely to function after viral internalization to promote viral uncoating or entry.

Therefore, it is suggested that the authors draw conclusions more specifically to the presented

data. In addition, it is recommended to show the structural/domain organization of LGMN so that the biological relevance of the point mutations can be better understood.

RE: We thank the reviewer for these insightful comments regarding our conclusions about LGMN's function. We agree that our initial conclusions don't accurately reflect the evidence provided in this section. We have now altered our analysis in this section in the following ways:

- We have removed mention of LGMN being ACE2-dependent from this section.
- We have removed mention of LGMN acting in a similar fashion to or being able to replace TMPRSS2 and instead emphasize that our results demonstrate that LGMN can promote infection in cellular contexts with or without TMPRSS2 expression.
- To better understand the potential localization of LGMN with Spike protein, we use confocal microscopy to image HEK293FT WT and ACE2OE cells with and without LGMN expression, when transduced with Spike-psuedotyped lentivirus (**new Fig. S10**). The experiments showed that SARS-CoV-2 Spike protein co-localizes with LGMN in the same cellular compartments, and that this colocalization is more pronounced in the context of ACE2 overexpression.
- Additionally, to address the role of localization of LGMN, we provide evidence that removal of LGMN's signal peptide can disrupt its infection promoting activity, but it can be rescued by re-targeting to cell surface membranes (**Fig 3h, Fig S11**). Taken together, this provides validation that the localization and protease activity are both important for its effects in relation to SARS-CoV-2.
- We have added structural information for LGMN (**Fig. 3f)** to better contextualize the relevance of the point mutations and domains to its catalytic activity and localization, respectively.

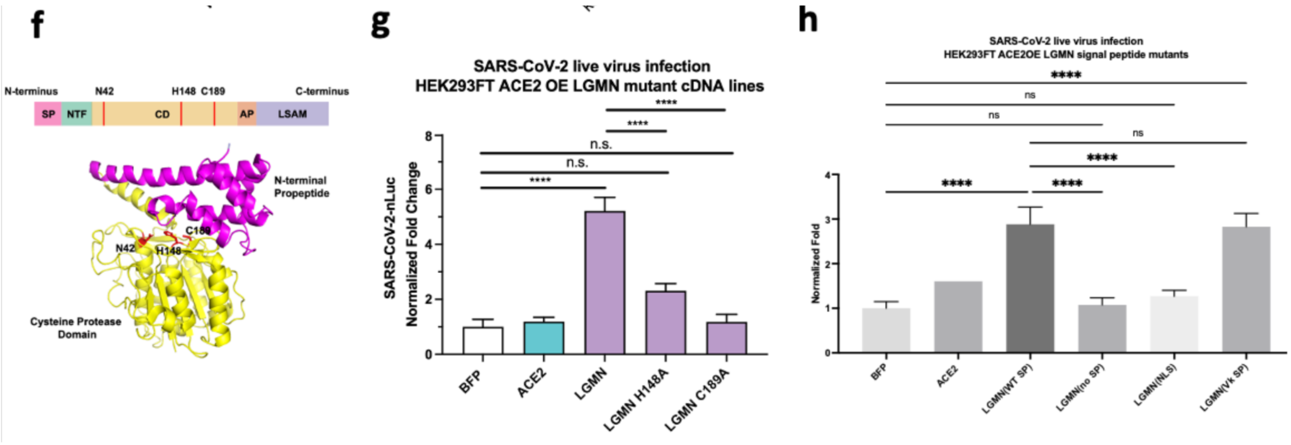

New Fig.3. Panel f, Structure and domains of human Legumain. SP: signal peptide, NTF: N-terminal fragment, CD: catalytic domain, AP: activation peptide, LSAM: legumain stabilization and activity modulation domain.**Panel g-h,** SARS-CoV-2 live virus infection of ACE2OE 293FT cells expressing LGMN or LGMN catalytic **(g)** and signal peptide **(h)** mutant cDNA. BFP is negative control (over-expressing BFP). **For panel h: WT SP**: LGMN with wild-type signal peptide; **no SP**, LGMN without any signal peptide; **NLS**, LGMN with no signal peptide and instead was fused to nuclear-localization signal

(NLS); **Vk SP**, LGMN with antibody kappa light chain signal peptide for targeting to cell surface. Results are normed to BFP control.

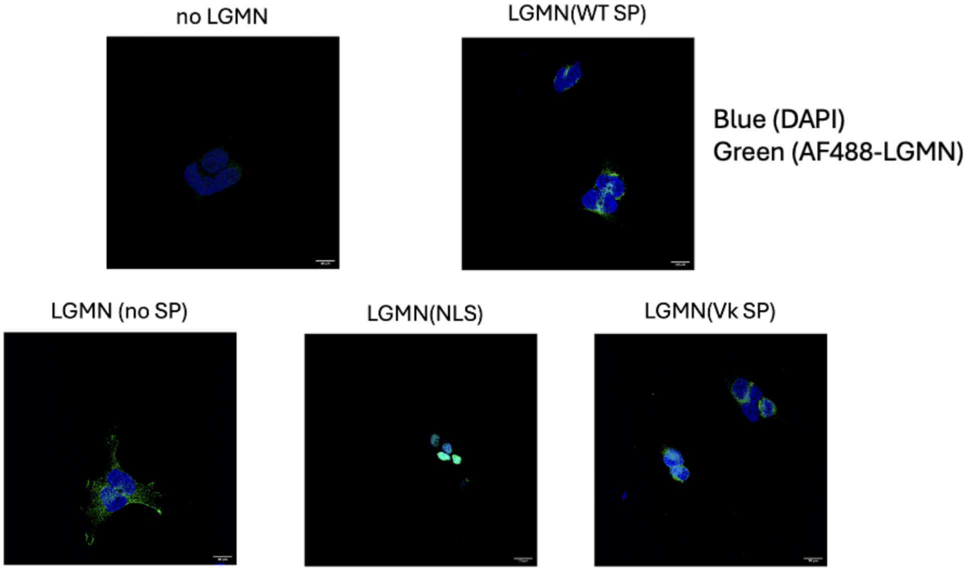

New Supp. Fig. 11. Imaging of LGMN signal peptide mutant localization. 293FT ACE2 OE cells transduced with LGMN or LGMN signal peptide variants (**same as above in Fig. 3h**). Cells were stained by immunofluorescence assay and imaged with a confocal microscope. **Blue**: DAPI, **green**: LGMN protein (with monoclonal LGMN antibody). Scale Bar: 10 μm.

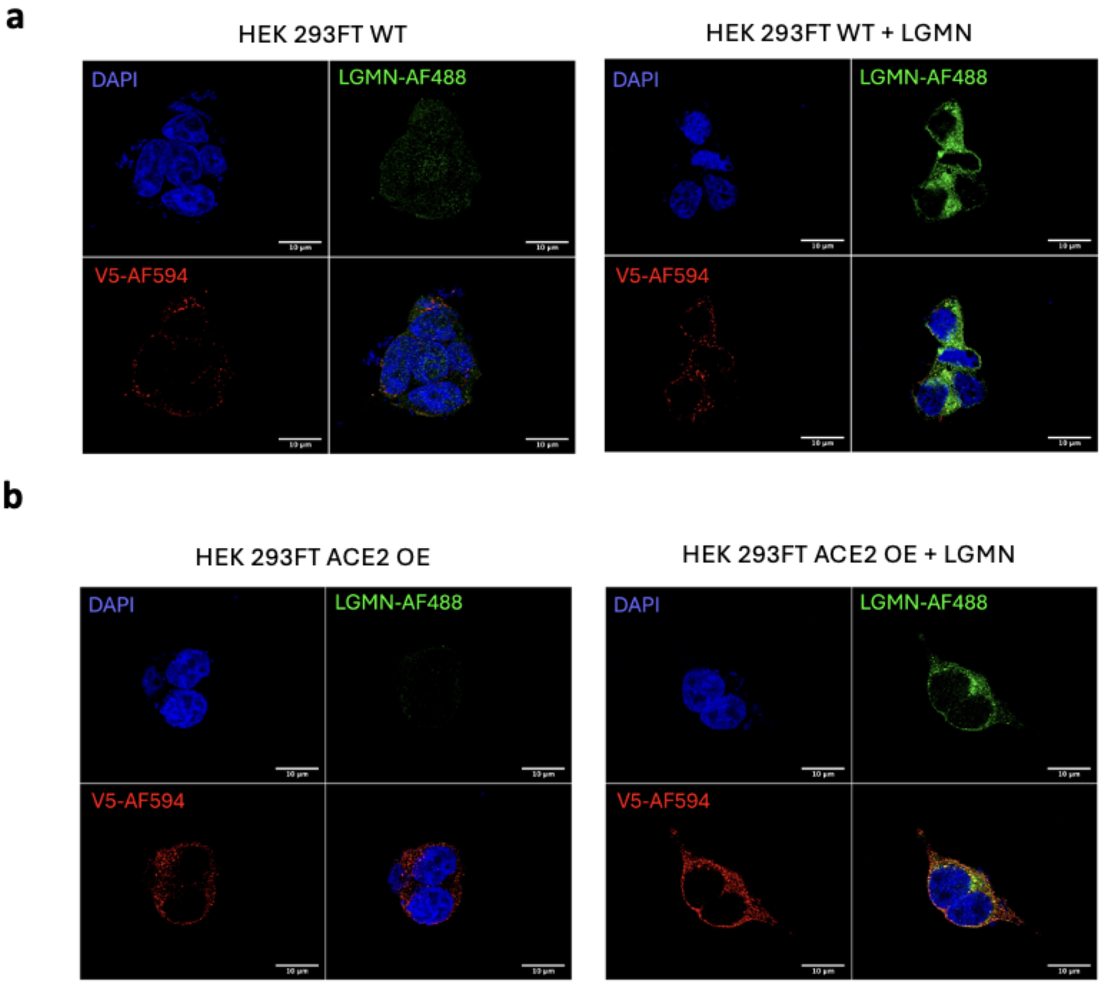

New Supp. Fig. 10. Confocal microscopy imaging of SARS-CoV-2 psuedovirus infection. a-b, 293FT WT **(a)** or ACE2 OE **(b)** cells with or without exogenous LGMN expression were transduced with Spike-V5tag pseudotyped lentivirus, then stained by immunofluorescence assay imaged with a confocal microscope at 4 hr post-transduction. Blue: DAPI; green: LGMN protein; red: Spike-V5 protein. Scale: 10 μm.

4. Fig 5. Is there any specific reason for the authors to switch the cell line between 293T and 293FT in this figure?

RE: We tried to produce ACE2 KO in the 293FT cell line, but had issues getting viable single cell clones. As 293FT cells are derived from the parental 293T lines, we opted to obtain a commercially available line ACE2-KO 293T line to answer our questions regarding the role of ACE2 expression in KCNA6 and LGMN infection promoting activity. This line is clonally selected and validated by sanger sequencing to have full biallelic knockout. We used 293FT cells in all other experiments to maintain consistency with screening conditions.

Minor:

1. It is recommended that the quality of the constructed CRISPR libraries are described.

RE: We have now included a figure detailing the CRISPR libraries' quality, including skew ratio/dropout and other library information (New Supp. Fig. S2) to go along with the fill library sequences (Supplementary Table 1).

A

Metric	Value
Total Reads Processed	6,308,415
Perfect Guide Matches	5,810,600
Nonperfect Guide Matches	258,901
Perfect Guide Match Rate	95.7%
Undetected Guide Rate	0.6%
Top 10% to Bottom 10% Skew Ratio	2.42

B

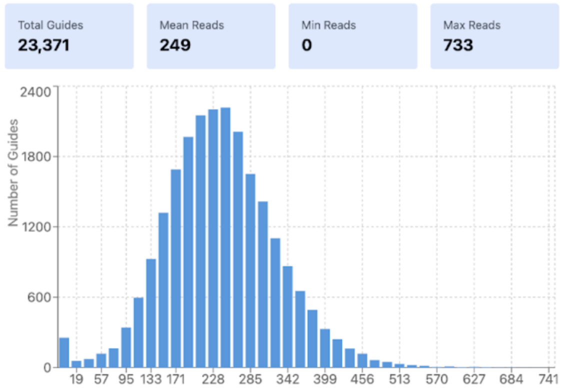

New Supplementary Fig. 2. Membrane-wide CRISPRa plasmid library sequencing analysis. a, Statistics for sequencing of membrane-wide CRISPRa plasmid library. b, Distribution of gRNAs and representation in the membrane-wide CRISPRa plasmid library.

2. P6 line35, the description was Fig 3d instead of Fig 3e.
3. P6 line41, the description was Fig 3e instead of Fig 3d.

RE: We have corrected the figure references. Thank you very much for your help on improving our manuscript.

Reviewer #2: In this manuscript, Dinesh et al. report multiple CRISPR activation screens to identify genes that promote SARS-CoV-2 infection. Although several other groups have already published such screens, publishing additional CRISPR screen results may still have added value, as the outcomes of screens performed in different labs are quite variable. After performing preliminary validation experiments on the top-ranked genes, the authors focus on the two genes LGMN and KCNA6. Although it is not clear why these two factors were chosen for further studies, the authors assess the effect of LGMN/KCNA6 overexpression, knockout, and pharmaceutical inhibition on SARS-CoV-2 infection and the results of these validation experiments are sufficiently convincing. It appears that no other publications describe extensive validation of these host factors. Finally, the authors check whether LGMN/KCNA6 overexpression influences the expression levels of other known SARS-CoV-2 host factors and

evaluate the expression of the two genes in disease-relevant tissues. Although some aspects of the manuscript are somewhat unclear/confusing, the main conclusions are relevant, sufficiently novel and are well supported by the data. However, the authors must address the following major issues before the study is suitable for publication in PLoS Pathogens

RE: We thank the reviewer for positive comments and constructive feedback that help to improve our work.

Reviewer #3: In the manuscript by Dinesh et al, the authors performed a membrane-wide CRISPRa screen to identify critical host factors for SARS-CoV-2 entry. The findings in this manuscript are informative and provide new insights into host-directed therapeutic targets for SARS-CoV-2. However, while the presented study is informative, the authors need to provide more mechanistic insights into how LGMN or KCNA6 promote SARS-CoV-2 entry.

RE: We thank the reviewer for positive comments and constructive feedback that help to improve our work.

Part II – Major Issues: Key Experiments Required for Acceptance
Please use this section to detail the key new experiments or modifications of existing experiments that should be absolutelyrequired to validate study conclusions.

Generally, there should be no more than 3 such required experiments or major modifications for a "Major Revision" recommendation. If more than 3 experiments are necessary to validate the study conclusions, then you are encouraged to recommend "Reject".

Reviewer #1: (No Response)

Reviewer #2: • The authors performed a validation experiment with a subpool CRISPRa library targeting top-ranked genes from their initial screen. However, discussion about the design and outcomes of this experiment are less than the bare minimum. Since this is a validation screen, I would expect a statement (and data) about how many of the 523 and 542 genes could be validated. Instead, the authors only mention that their top-ranked sgRNAs perform consistently, irrespective of the use of WT or D614G Spike. The authors should present and discuss the outcomes of this validation screen more clearly or, in case their results were disappointing, consider removing this experiment from the paper altogether, as its value appears to be very limited.

RE: We appreciate the reviewer's comment about the validation screen. After careful consideration, we have decided to remove this part from the paper as suggested, since the key findings are validated individually and this sub-screen doesn't add substantial new insights compared to the original screen.

• Figure 4d: In this experiment, the authors used three individual sgRNAs per gene to generate three separate knockout cell lines that are infected with virus. This strategy is common practice in CRISPR screen validation because testing multiple sgRNAs reduces the chance that the observed effects are due to off-target editing. Demonstrating that three unique sgRNAs all reduce virus infection would convincingly show that this is really due to on-target knockout. However, the authors aggregated data of the different guides for each gene and present only the mean of the guides. Data for each sgRNA should be presented separately as this is one of the most important figures in the paper and it should be as convincing as possible.

RE: We thank the reviewer for the suggestion regarding the presentation of knockout data. We have now provided the detailed multiple gRNA data in a new Supplementary Figure (**new Supp. Fig. S9**), showing individual results for each sgRNA separately.

• Page 6, lines 1-2: the authors conclude that CD7, KCNA6, and LGMN overexpression promote infection, but an effect of CD7 overexpression is not shown in Figures 2c and d. This should be corrected. Also, the authors may consider to adjust the phrase “similarly promote infection” as it suggests that effect sizes are the same for the different genes.

RE: We have corrected the text regarding CD7 overexpression effects. Thank you very much for the helpful comments.

• Figure 5a: The authors conclude from this figure that the stimulating effect of LGMN/KCNA6 overexpression on infection is dependent on ACE2. However, it is not clear whether the ACE2-KO cells are still infected by the virus. If ACE2-KO cannot be infected, it is quite unsurprising that overexpression of other genes will not have an effect but this would not prove that their functions depend on ACE2. Therefore, the authors should either show that the cells are infected, or remove the conclusion about dependency of LGMN/KCNA6 on ACE2.

RE: We appreciate the reviewer's insight regarding the wording used to describe the conclusions in this section of the manuscript. As ACE2 expression rescues the effect of ACE2 KO, but neither LGMN or KCNA6 are capable, we have revised the text to more precisely state that LGMN and KCNA6 are capable of promoting infection only in cellular contexts of ACE2 expression. Thank you for your comments.

• Figure 5d: The authors performed transcriptome analysis on LGMN/KCNA6 overexpressing cells but it is not clear to me what exactly is shown in the figure. The experiment is hardly explained in the text, it is not clear whether labels at the bottom represent knockout or overexpression, and the color scale is not labelled. This needs to be clarified.

RE: We have clarified the presentation of the transcriptome analysis in **Figure 5d**, in the updated figure:

- Added explanations in the text
- Clarified labels to indicate knockout vs. overexpression
- Added proper labeling for the color scale

Reviewer #3:

1.The authors have validated several candidates by cDNA overexpression and observed minor phenotype following pseudovirus and authentic SARS-CoV-2 infection. However, most of the top hits were not validated. The rationality for choosing the candidates for validation is not explained in the manuscript. Notably, LGMN or KCNA6 is not even the positive hits in 293T-ACE2 cells screens. (KCNA rank in different condition screens (#68, #309，#3960,#3350).

RE: We thank the reviewer for requesting clarification about our candidate selection process. We have revised the text to provide an explanation for the selection of our candidate genes (lines 33-36). In brief, due to the noisiness of CRISPRa screens we selected genes primarily based on overlaps analysis between screens (top 10% for each screen, but not found in VSVG control screens), representation in enriched pathways, and enrichment in disease-relevant tissues.

- Analysis of screen noise levels
- Overlapping analysis between different conditions
- Pathway analysis
- Expression patterns in disease-relevant tissues (lung, respiratory and GI tracts, immune cells)
- Based on expression in tissues known to be involved in SARS-CoV-2 pathology

2.The authors compared several LOF screen results with their GOF screen findings. However, the authors haven't done this comparasion with previous GOF screens (such as PMID: 35879413, 35879412 etc). Is there any hits overlap between this study and previous GOF screens?

RE: We have now included additional overlapping analysis with other CRISPRa GOF screening studies (**Updated Supp. Fig. S3,** the newly added **"CRISPR activation screens"** part). Thank you very much for the helpful comment.

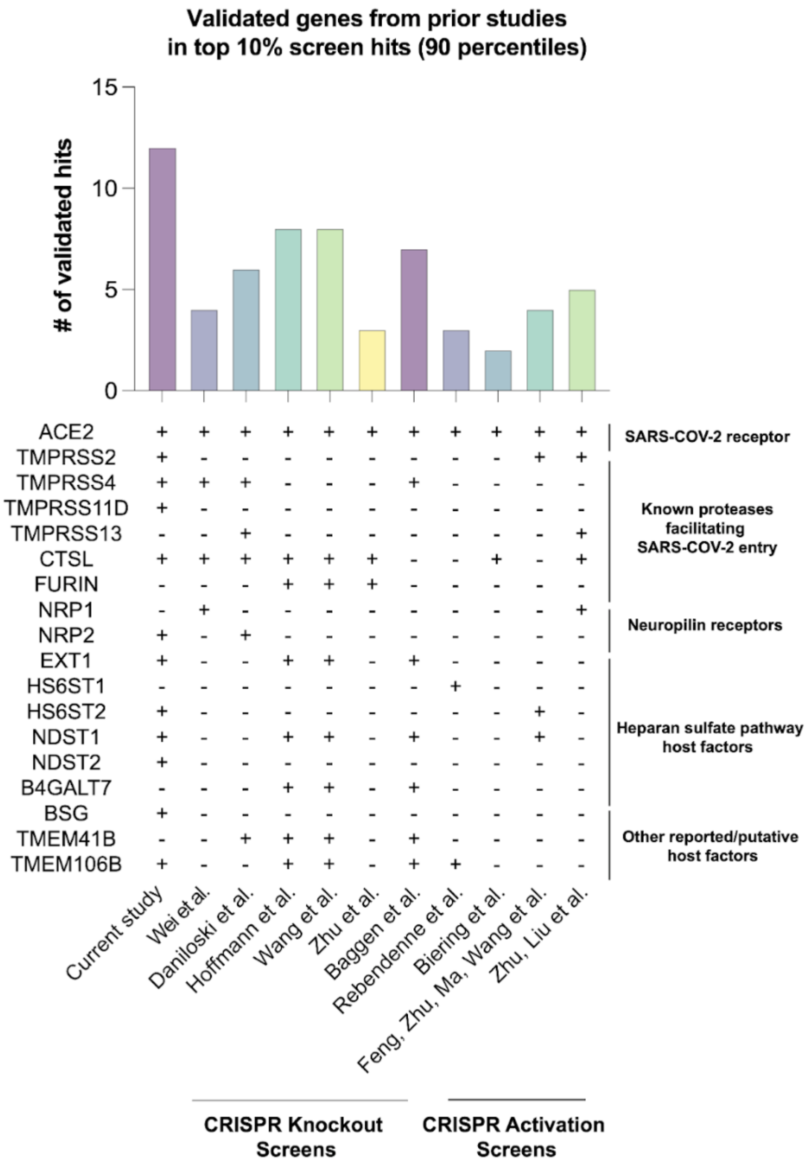

3.The cell lines used for SARS-CoV-2 infection is not well characterized cell lines for CoVs infection. Do these hits identified in this study have similar phenotype in cells most used for CoV infection such as in Vero, Calu-3?

RE: Regarding the use of additional cell lines 1) we focused on cell lines where our top hits were endogenously expressed to enable knockout experiments at physiological expression levels. This approach helps address concerns about artificial expression levels in the initial GOF approach, and 2) A primary aim of this manuscript was finding genes that drive infection in tissues/contexts not well studied with current experimental systems. While we acknowledge the importance of well-established lines like Vero and Calu-3, we believe our current focus provides valuable insights into SARS-CoV-2 across tissues.

4.The authors didn't provide more molecular mechanisms by which LGMN or KCNA6 promote viral entry. Does protease LGMN promote viral entry by cleaving and activating Spike protein like TMPRSS2?

RE: We appreciate the reviewer's request for additional mechanistic insights. We have strengthened this aspect by:

- Demonstrating that LGMN's protease catalytic residues are required for enhancing SARS-CoV-2 infection (**Fig 3f-g**)
- Adding new data showing the importance of membrane targeting and signaling peptide removal/mutation for LGMN's function (**new Fig. 3h**), with corresponding imaging study via confocal microscopy to validate the localization of these LGMN mutants (**New Supp. Fig. 11**).
- Adding new data showing that LGMN and Spike protein (with pseudoviral infection) co-localize in the same cellular compartments, and that this colocalization is more pronounced in the context of ACE2 overexpression (**New Supp. Fig. 10**) .

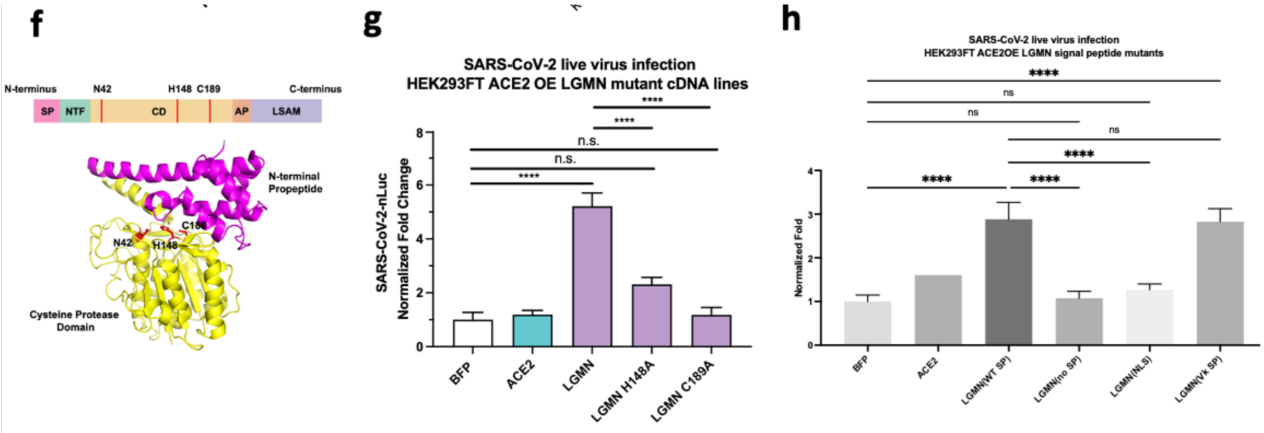

New Fig.3. Panel f, Structure and domains of human Legumain. SP: signal peptide, NTF: N-terminal fragment, CD: catalytic domain, AP: activation peptide, LSAM: legumain stabilization and activity modulation domain.**Panel g-h,** SARS-CoV-2 live virus infection of ACE2OE 293FT cells expressing LGMN or LGMN catalytic **(g)** and signal peptide **(h)** mutant cDNA. BFP is negative control (over-expressing BFP). **For panel h: WT SP**: LGMN with wild-type signal peptide; **no SP**, LGMN without any signal peptide; **NLS**, LGMN with no signal peptide and instead was fused to nuclear-localization signal (NLS); **Vk SP**, LGMN with antibody kappa light chain signal peptide for targeting to cell surface. Results are normed to BFP control.

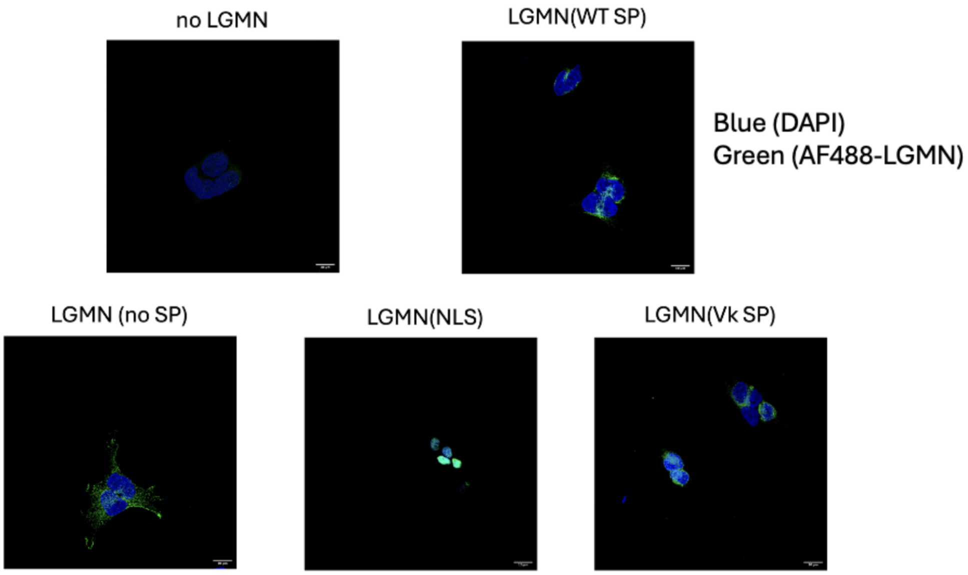

New Supp. Fig. 11. Imaging of LGMN signal peptide mutant localization. 293FT ACE2 OE cells transduced with LGMN or LGMN signal peptide variants (**same as above in Fig. 3h**). Cells were stained by immunofluorescence assay and imaged with a confocal microscope. **Blue**: DAPI, **green**: LGMN protein (with monoclonal LGMN antibody). Scale Bar: 10 μm.

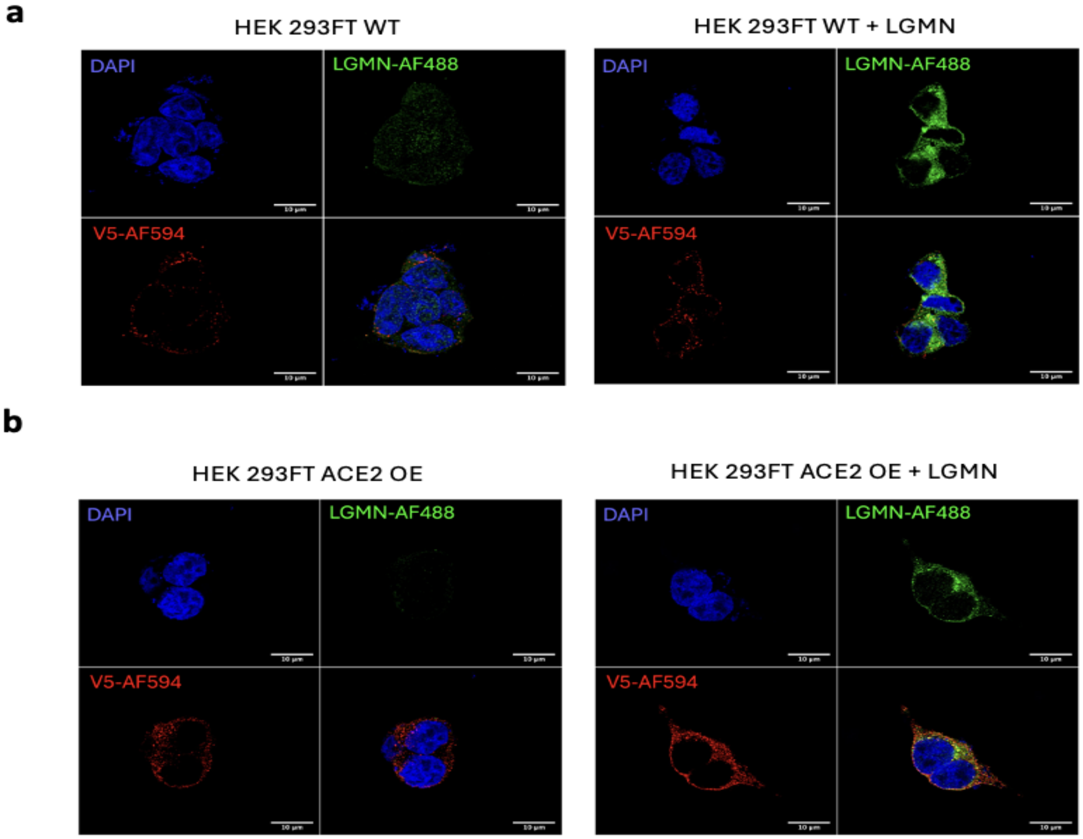

New Supp. Fig. 10. Confocal microscopy imaging of SARS-CoV-2 psuedovirus infection. a-b, 293FT WT **(a)** or ACE2 OE **(b)** cells with or without exogenous LGMN

expression were transduced with Spike-V5tag pseudotyped lentivirus, then stained by immunofluorescence assay imaged with a confocal microscope at 4 hr post-transduction. Blue: DAPI; green: LGMN protein; red: Spike-V5 protein. Scale: 10 μm.

We acknowledge that further mechanistic studies, particularly for KCNA6, will be valuable for future work. We hope that this investigation on KCNA6 could be for a future study.

Part III – Minor Issues: Editorial and Data Presentation Modifications
Please use this section for editorial suggestions as well as relatively minor modifications of existing data that would enhance clarity.

Reviewer #1: (No Response)

Reviewer #2:
• Page 3, lines 35-36: This sentence suggests that inhibitors of viral genome replication are in general less effective that entry inhibitors, but many effective replication inhibitors exist for other viruses. The authors should consider weakening this statement.

RE: We that the reviewer for the note, We have modified the statement about inhibitors of viral genome replication on Page 3, lines 35-37 to better reflect that our statement pertains to SARS-CoV-2 specifically and not viruses more broadly.

• Page 4, lines 18-19: "We then confirmed that these cells were capable of sgRNA-directed gene activation". It is not clear how it was shown that sgRNA-directed gene activation is working, as Figure S1c appears to only show that the cells can be infected.

RE: We thank the reviewer for catching this error. We have added the appropriate data showing the transfection experiment that confirmed gene activation potential in our cell lines (Fig S1d).

• Page 4, line 39: It is specifically mentioned that the previously described receptor NRP1 is found in the screens, with a reference to Figures 1d-e, g. However, this gene is not shown in the figures.

RE: We have removed the reference to NRP1 in the figures.

• References to Figures 3d and 3e appear to be swapped.

RE: We thank the reviewer for the note, the figure has been corrected.

Reviewer #3: The study would benefit from more robust virologic assays. The infection experiments utilize pseudovirus assys and luciferase as a read out. A more direct measure of viral replication or susceptibility would be of value. This would be ideally done by plaque assay.

RE: We thank the reviewer for the comments. However, while we recognize the value of plaque assays, we should note that 1) nanonluciferase assay has been previously validated as a measure of live replication-competent SARS-CoV-2 infection by other groups (https://pubmed.ncbi.nlm.nih.gov/32854108/, https://pubmed.ncbi.nlm.nih.gov/32526206//) and 2) reproducing our major findings in this manuscript with new assays given the constrained nature of running BSL-3 experiments would be time, resource, and labor intensive. In an effort to maintain uniformity in our data, we have performed new experiments using the nanoluciferase SARS-CoV-2. We hope the reviewer understands our current limitations.

We thank the reviewers again for their constructive feedback, which has significantly strengthened our manuscript. All changes are highlighted in the revised text, and new data are incorporated into Figures 2-6 and supplementary materials.
